# Advancing remote photoplethysmography (rPPG) to facilitate cardiac monitoring in naturalistic settings using webcam technology

**DOI:** 10.3758/s13428-026-02953-x

**Published:** 2026-04-23

**Authors:** Sascha P. Woelk, Sarah N. Garfinkel, Lewend Mayiwar, Klemens M. Knöferle

**Affiliations:** 1 Institute of Cognitive Neuroscience, University College London, Alexandra House, 17-19 Queen Square, London, WC1N 3AZ England; 2 Oslo Business School, Oslo Metropolitan University, Oslo, Norway; 3 Department of Marketing, BI Norwegian Business School, Oslo, Norway

**Keywords:** Remote photoplethysmography (rPPG), Imaging photoplethysmogram, Camera-based photoplethysmography, HR variability (HRV), Pulse rate variability (PRV), Online experiments, Remote monitoring, Benchmark

## Abstract

Remote photoplethysmography (rPPG) is a technique for the monitoring of cardiac signals using simple consumer-grade cameras, such as webcams. Its cost-effectiveness and accessibility make it a promising tool for widespread application, particularly in remote and resource-limited research settings. However, it remains unclear whether rPPG can accurately capture the moment-to-moment fluctuations in heart rate (HR) required for robust HR variability (HRV) estimation. In this study, we collected a novel dataset of 77 participants who completed three behavioral tasks under controlled laboratory conditions, designed to mirror the recording setup typically encountered in home-based online experiments. We validated four rPPG pipeline configurations against the electrocardiogram (ECG) ground truth, using a comprehensive set of measures capturing both signal quality and cardiac variability. Moreover, using data from an emotion anticipation task, we assessed whether, in a typical experimental setting, analyses based on rPPG data yielded statistical results comparable to those obtained from ECG. Our results indicate that rPPG is a promising tool for detecting HR and HRV at the group level, particularly in high-powered studies. At the individual level, rPPG reliably captures average HR, but the accuracy of individual-level HRV estimates remains limited. The findings underscore rPPG’s potential as a low-cost, noninvasive research tool, while highlighting the importance of managing its limitations such as sensitivity to excessive movement and poor illumination through thoughtful study planning and execution.

**Supplementary Information:**

The online version contains supplementary material available at 10.3758/s13428-026-02953-x.

## Introduction

### Background

Cardiac data and cardiac metrics such as heart rate (HR) and heart rate variability (HRV) are widely used in the social and medical sciences as well as in clinical practice to assess constructs as diverse as physical health and autonomic functioning (Koch et al., 2019; Shaffer & Ginsberg, 2017), mental health and affective processes (Beauchaine & Thayer, 2015; Mulcahy et al., 2019), and stress (H.-G. Kim et al., 2018). The growing field of interoception research, which examines how the nervous system senses, interprets, and integrates signals from within the body (Khalsa et al., 2018), has moreover shown that cardiac processes play a wider role in cognition (Critchley & Garfinkel, 2018), including in self-perception and agency (Aspell et al., 2013; Craig, 2009; Koreki et al., 2022; Sel et al., 2017), social cognition (Azevedo et al., 2017; Moeini-Jazani et al., 2017), visuo-tactile experience (Al et al., 2020), learning and memory (Pfeifer et al., 2017), pain sensitivity (Gray et al., 2009), and emotion (Garfinkel et al., 2021; Garfinkel & Critchley, 2016).

The electrocardiogram (ECG) is regarded as the gold standard for assessing cardiac activity. Each heartbeat is initiated by an electrical impulse that travels through the cardiac tissue and triggers coordinated contraction of the heart. The ECG directly measures the electric potential difference generated by this depolarization and subsequent repolarization (Hall & Hall, 2021; Laborde et al., 2017; Malik et al., 1996; Shaffer & Ginsberg, 2017). However, the cost and operational complexity of ECG systems limit their use to specialized laboratories with trained researchers (Faruk et al., 2021).

An alternative method for collecting cardiac data is photoplethysmography (PPG) (Gil et al., 2010; Schäfer & Vagedes, 2013). In contact-PPG (cPPG), a light-emitting diode placed in direct contact with the skin shines invisible infrared light into the tissue, and a photodetector measures the amount of light that is backscattered. Because blood absorbs light more than the surrounding tissues, the measured light corresponds to the blood-volume-pulse (BVP), that is, the pulsatile wave of blood traveling from the heart through the blood vessels beneath the skin. Since its discovery in the 1930 s, cPPG has become commonplace in the medical field, where it is widely used to measure cardiovascular parameters, including blood oxygenation, cardiac output, and blood pressure (Elgendi, 2012). More recently, cPPG has also become a key sensing technology for wearables such as smartwatches and fitness trackers, where it is used to estimate physiological parameters, including stress, sleep quality, and exercise (Charlton et al., 2023).

In the early 2000s, research interest in BVP detection via the visible light spectrum increased, and it was shown that the BVP signal could be measured remotely by analyzing a video of the human face, recorded under ambient light using simple consumer-grade RGB cameras at frame rates as low as 25 Hz (Béres & Hejjel, 2021; Verkruysse et al., 2008). Remote photoplethysmography (rPPG) relies on the fact that the strength of the BVP signal differs across the color spectrum, with the green channel exhibiting the strongest signal due to greater absorption of green light by hemoglobin (Verkruysse et al., 2008). Moreover, the amount of backscattered light from the skin can be decomposed into distinct types of reflection: diffuse reflection (i.e., light that has traveled through the skin and consequently exhibits intensity variations due to subcutaneous blood flow), specular reflection (i.e., light directly backscattered from the epidermis, carrying no information about blood flow), and quantization noise of the camera sensor (de Haan & Jeanne, 2013; Verkruysse et al., 2008; W. Wang et al., 2017).

rPPG opens an exciting opportunity for noninvasive and cost-effective measurement of cardiac data in naturalistic research settings and remote online studies. Most previous rPPG studies have focused on average HR when comparing the accuracy of rPPG to the cPPG or ECG ground truth. HR reflects the average number of heartbeats within a given time window (e.g. 1 min). The results usually indicate strong agreement between methods, with differences in the range of 2–3% (approximately 1–2 beats per minute [BPM]) when participants are at rest (Dasari et al., 2021; Di Lernia et al., 2024; Haugg et al., 2022).

However, it remains unclear whether the accuracy of rPPG is sufficient to reliably capture HRV. HRV quantifies fluctuations in the intervals between consecutive heartbeats. HRV metrics are a useful index of neurocardiac autonomic function, reflecting the dynamic balance between sympathetic and parasympathetic activity (Shaffer & Ginsberg, 2017). Because accurate calculation of HRV metrics depends on fine-grained information about momentary changes in cardiac activity, it places substantially higher demands on signal precision than HR measurement (see Appendix C for details on HRV metric definitions).

Consequently, extracting reliable HRV metrics from rPPG is an active area of research. Several recent studies have proposed pipeline improvements and algorithm innovations. For example, Pai et al (2021) developed a post-processing pipeline which combines an automatic adaptive bandpass filter to reduce motion artifacts with pulse frequency demodulation for robust instantaneous HR extraction. In a small sample of 16 participants including different skin tones and motion conditions, their pipeline showed excellent agreement between HRV metrics from rPPG and cPPG. Similarly, Gudi et al. (2020) focused on refined post-processing to increase rPPG robustness to motion and illumination variability, combining head-pose-based motion suppression with two-step wide- and narrow-band frequency filtering. Extensive evaluation of their pipeline across 13 datasets demonstrated robustness to illumination variations, as well as moderate facial and body movements. Odinaev et al. (2023) introduced an HRV extraction algorithm based on the wavelet scattering transform technique, followed by adaptive bandpass filtering. It outperformed prior algorithms in three public datasets and an in-house dataset of 14 participants. More recently, researchers have also started to develop deep learning models focused on HRV extraction from rPPG. For example, Seq-rPPG (K. Wang et al., 2024) implements spectral decomposition within a convolutional neural network architecture, and was trained on short duration recordings. Another example is AttenHRVNet (Zhan et al., 2026), which is based on spatiotemporal feature extraction and was designed to improve HRV measurement during long-term recordings.

Collectively, these studies demonstrate substantial scientific progress in the application of rPPG to HRV measurement. However, most validations rely on pre-existing tightly controlled laboratory datasets or small in-house samples which lack benchmarking using established rPPG algorithms, thus limiting generalizability. The notable exception is a recent paper by Wang et al. (2024), who collected a relatively large novel dataset (*N* = 58 participants) under remote learning conditions and used it to evaluate the performance of their novel deep learning rPPG algorithm relative to two established rPPG algorithms. Apart from this, only a handful of studies have systematically assessed rPPG accuracy in terms of HRV metrics: McDuff et al. (2014) measured HRV in a sample of 10 participants at rest and during a mental arithmetic task and found very high correlations for both high-frequency HRV (HF-HRV) and low-frequency HRV (LF-HRV) between the rPPG and cPPG ground truth. Tohma et al. (2021) evaluated how varying illumination levels, camera frame rates, and body movement affect the accuracy of HRV metrics from rPPG, with a focus on telemedicine. In a small sample of three participants, they found that best results were obtained with illumination levels of 500–700 lux, cameras with frame rates of at least 30 frames per second (fps), and limited head movement. In a more recent, larger benchmark study, van Es et al. (2023) evaluated eight rPPG algorithms using a publicly available dataset of 42 participants. Of these, only two algorithms yielded moderate to strong correlations with time-domain HRV metrics, whereas results for frequency-domain HRV metrics were generally unreliable.

Together, these findings highlight the need for systematic evaluation of rPPG accuracy for HRV measurement in larger samples with controlled yet ecologically valid recording conditions.

### The current study

Building on these insights, the goal of the current study was to provide a structured evaluation of rPPG accuracy for HRV measurement under controlled yet ecologically valid research conditions. A sample of 77 participants completed the experiment in the laboratory, with the setup purposefully designed to mirror the technical and recording environment encountered in home-based online experiments, including webcam placement, frame rate, and illumination. Video data were collected via an online survey platform and processed securely in the cloud. Electrocardiography (ECG) was recorded simultaneously with video and served as the gold-standard reference for all cardiac measures. Unlike most existing datasets, which consist of short, single-condition recordings, this novel dataset comprises three behavioral paradigms with multiple trials—an emotion anticipation task, a heartbeat counting task, and a time counting task—providing a diverse and ecologically relevant range of autonomic responses. In total, the dataset comprises 2,436 videos and associated ECG recordings.

In the present manuscript, we report HR and HRV validation based on the recordings from all tasks, alongside behavioral analyses from the emotion anticipation task. We focused our benchmark on the widely used plane-orthogonal-to-the-skin algorithm (POS, W. Wang et al., 2017), which has consistently demonstrated state-of-the-art performance in prior benchmarks. Specifically, we evaluate POS in its raw form and with pulse frequency demodulation (HRVCam; Pai et al., 2021), each tested in both holistic and patch-based configurations. The most effective pipeline configuration has been made publicly available on OSF to support its use in future research.

## Methods

### Experimental design

#### Participants

One hundred three adults aged 18–65 years with normal or corrected-to-normal vision and without any other exclusion criteria were recruited from the participant pool of BI Norwegian Business School. All participants provided written informed consent for facial video and heartbeat recordings, as well as for general data retention and analysis. The study received approval from the BI Research Ethics Committee of the BI Norwegian Business School and complied with the ethical standards summarized in the Declaration of Helsinki.

Six participants were excluded due to missing data and data entry errors. In addition, data from 20 participants were excluded following independent ECG quality control, as the signal quality was insufficient to serve as a reliable reference for rPPG benchmarking. To ensure that these exclusions did not introduce systematic bias, we compared participant and recording characteristics between retained and excluded participants. Excluded participants had comparable height, *t*(31) = 1.4, *p* =.18, but lower body weight, *t*(45.7) = 2.9, *p* =.006, resulting in lower body mass index (BMI), *t*(46) = 2.7, *p* =.009. Moreover, a proportionally larger number of male participants were excluded (Fisher’s exact, *p* =.006). This pattern suggests poorer ECG performance in leaner males, potentially due to greater chest hair density, which is known to significantly increase electrode impedance and reduce adhesion (Ashley & Niebauer, 2004; Tian et al., 2023; L. Yang et al., 2022a, 2022b). No other statistically significant differences were observed in age, *t*(26) = −0.7, *p* =.50, skin tone, *t*(30.1) = −1.5, *p* =.139, physical activity level, *t*(30.7) = −0.3, *p* =.79, participant movement during video recordings, *t*(33.7) = −1.7, *p* =.10, or average video illumination, *t*(35.3) = −1.6, *p* =.11. Full results of this comparison are reported in Appendix A.

The final sample comprised 77 participants. We conducted a post hoc power analysis to validate that this sample size was sufficient to reliably assess equivalence between rPPG and ECG in our key outcome measure—momentary changes in HR. The analysis focused on mean absolute error (MAE) for two standard time-domain HRV metrics: the standard deviation of normal-to-normal intervals (SDNN) and the root mean square of successive deviations (RMSSD). A detailed definition of both metrics is included in Appendix C. The smallest effect size of interest (SESOI) was defined as ±15 ms for both SDNN and RMSSD, based on known biases of short-term HRV metrics relative to longer recordings (Munoz et al., 2015), as well as typical individual differences in short-term HRV metrics observed across sex, ethnicity, and cardiovascular risk factors (O’Neal et al., 2016; Voss et al., 2013, 2015). Using the observed MAE values from our best-performing rPPG pipeline (**|∆** SDNN**|** = 11.45 ± 14.34 ms; **|∆** RMSSD**|** = 11.02 ± 10.43 ms), one-sample equivalence tests (TOST, α =.05) indicated statistical power of 69% for SDNN and 95% for RMSSD. Accounting for multiple recordings per participant (mean = 9; ICC = 0.5) increased these estimates to 89.5% and 99.8%, respectively. The sample demographics are summarized in Table 1.

**Table 1:** Sample summary characteristics

*(N* = 77)	***M***	***SD***
**Age (years)**	24	3.89
**Weight (kg)**	69.44	15.85
**Height (cm)**	170.88	9.67
**BMI (units)**	23.63	4.10
**Sports (times per week)**	2.41	1.52
**Female (%)**	44.2	-
**Male (%)**	54.5	-
**Other gender (%)**	1.3	-

#### Procedure

All participants attended the experiment in person, one-by-one, in a well-lit testing room at BI Norwegian Business School. Throughout the experiment, participants were seated in front of a computer with a Trust GXT 1160 webcam (Trust, 2025) attached to the top of the computer screen.

The entire experiment was programmed in the online survey tool Qualtrics (see OSF repository for questionnaire file). Video recording was integrated in the Qualtrics survey via the JavaScript integration of the browser-based video recording tool AddPipe (Pipe Services S.R.L., 2025). All videos were stored on encrypted Amazon AWS S3 cloud storage (Amazon Web Services, Inc., 2025b) and processed on an Amazon AWS EC2 instance (Amazon Web Services, Inc., 2025a) using AWS SageMaker Jupyter Notebooks (Amazon Web Services, Inc., 2025c). This setup was designed under the guidance of Amazon AWS solution architects and internally reviewed for data management and protection by BI Norwegian Business School’s Digital Department, Data Protection Officer, and Chief Privacy Officer, and externally by Sikt (the Norwegian Agency for Shared Services in Education and Research).

All participants completed three computerized tasks while being filmed. For all tasks, participants were asked to sit comfortably, breathe normally, limit movement, and face the webcam on top of the computer screen. Unless otherwise specified, all tasks were conducted under realistic illumination conditions with fluorescent ceiling lights set to regular brightness.

### Emotion anticipation

The emotion anticipation task employed a computerized two-stimulus paradigm to assess participants’ cardiac response in anticipation of emotional stimuli (Poli et al., 2007). The stimuli included the emotion categories “Object,” “Injury,” and “Erotica.” For each category, a total of 50 pictures were selected from the International Affective Picture System (IAPS; Lang et al., 2005). For each participant, a random subset of six pictures per emotion category was drawn, resulting in a total of 18 trials. The order of stimulus presentation was fully randomized. On each trial, first the name of the emotion category (i.e., “Object,” “Injury,” or “Erotica”) was displayed on the screen for 1,500 ms. Then, after a 4,500-ms interval, a picture from the corresponding emotion category was displayed for 2,000 ms. Each trial lasted 16 s. Participants were instructed to focus on the computer screen and attend to both words and pictures. After each trial, participants rated their emotional experience in terms of valence (unpleasant–pleasant) and arousal (calm–excited) using a five-point visual analogue scale.

The remaining two tasks were a heartbeat counting task (Schandry, 1981), following a six-trial procedure outlined by Garfinkel et al. (2015), under both regular and dimmed lighting conditions (12 trials in total, grouped in two counterbalanced sets of six trials under regular and dimmed lighting, respectively), and a time estimation task (Desmedt et al., 2020) comprising six trials with duration matched to the heartbeat counting task. For the current manuscript, behavioral analyses from these two tasks were omitted due to the study scope, and only video recordings from these tasks were included in the analysis of rPPG accuracy, maximizing statistical power for HR and HRV analyses.

### Questionnaires

After the experimental tasks, participants completed the Interoceptive Accuracy Scale (Murphy et al., 2020), the Patient Health Questionnaire-9 (Kroenke et al., 2001), the short form of the Rational-Experiential Inventory (Pacini & Epstein, 1999), and the trait version of the State-Trait Anxiety Inventory (Spielberger et al., 1983), in randomized order. Questionnaires were excluded from the current manuscript, as the focus was on validation of the physiological measures.

In addition, all participants provided information about age, gender, height, weight, and level of physical activity (8-point Likert scale, ranging from “Never” to “More than four times per week”).

### Electrophysiological recordings

Throughout the experiment, ECG activity was recorded using two disposable, pre-gelled spot electrodes (Ambu Neuroline 720, Ambu A/S, Denmark). One electrode was placed below the left clavicle, and the other electrode was placed on the upper right chest. The ECG signal was amplified and recorded with a Sudologger 3 device (Biogauge A/S, Norway). The recordings were stored as flat files on the experimental computer and later aligned with the video recordings based on timestamps.

## Cardiac signal extraction from video

rPPG pre- and post-processing choices can significantly affect the quality of the extracted BVP signal. Therefore, we based our pipeline configuration on a previous benchmark study that had evaluated the performance of different pre- and post-processing configurations (Unakafov, 2018). All preprocessing steps, as well as the BVP extraction, were implemented using the Python framework for virtual HR (pyVHR; Boccignone et al., 2022).

### Video preprocessing

#### Face segmentation

The first step in every rPPG pipeline is the extraction of facial skin regions from each video frame. Previous research has shown that rPPG performance can differ based on the selected facial regions due to differences in skin thickness, microvasculature, and the amount of facial movement. Best performance is generally observed either when the entire face is chosen as region of interest (holistic ROI approach), or when rectangular patches from the forehead and cheeks are targeted (patches ROI approach) (Boccignone et al., 2020; D.-Y. Kim et al., 2021; Kwon et al., 2015). We implemented both approaches for comparison.

For the patches approach, we selected a collection of partially overlapping patches measuring 40 × 40 pixels that evenly covered the forehead and both cheeks. The specific patch locations were selected using the landmarks provided by the computer vision library MediaPipe Face Mesh (Kartynnik et al., 2019). A visual illustration of the landmark locations and patch sizes is included in Appendix B.

#### Exclusion of non-skin elements

For both the holistic approach and the patches approach, the first processing step was to extract the portion of each video frame containing the face, using the MediaPipe computer vision library (Lugaresi et al., 2019).

Next, facial areas containing non-skin elements were excluded. We removed the eyes, mouth, and surrounding areas using convex hull masking. Areas containing other non-skin elements, such as glasses or facial hair, were filtered via red-green-blue (RGB) thresholding with an RGB value range set from 40 to 255.

Finally, the per-frame average RGB values were calculated. In the holistic approach, averaging was performed across the entire face, resulting in a single RGB time series. In the patches approach, averaging was performed within each 40 × 40 pixel patch, resulting in one RGB time series per patch.

No further preprocessing was applied before extracting the BVP signal.

### Blood volume pulse (BVP) extraction from video images

The next step in the rPPG pipeline was the extraction of the BVP signal from the RGB time series. For this, we chose the plane-orthogonal-to-the-skin algorithm (POS, W. Wang et al., 2017). Several studies and benchmarks have shown that POS generally outperforms other rPPG algorithms (Haugg et al., 2022; Unakafov, 2018; van Es et al., 2023; Zhu et al., 2023). Notably, it even outperforms deep learning and neural network approaches, being more robust to movement and lighting variations (Przybyło, 2022; Z. Yang et al., 2022a, 2022b; Zhu et al., 2023).

### BVP post-processing

#### Pipeline 1: Holistic ROI selection and time-domain HR extraction

As a baseline, we implemented a minimal post-processing pipeline with holistic ROI selection, followed by time-domain systolic peak extraction. Again, post-processing steps were based on previous benchmark results (Unakafov, 2018). The BVP signal obtained from the POS algorithm was moving-average-filtered with a window length of six frames and bandpass-filtered between 0.5 and 2.5 Hz using a third-order Butterworth filter.

To improve peak detection, the BVP signal was upsampled to match the ECG recording’s 330 Hz sampling frequency, using cubic spline interpolation. Finally, systolic peaks were detected using the event-related moving average with a dynamic threshold algorithm (Elgendi et al., 2013) as implemented in the physiological signal processing toolbox NeuroKit2, version 0.2.4 (Makowski et al., 2021).

#### Pipeline 2: Patches ROI selection and time-domain HR extraction

Pipeline 2 was identical to Pipeline 1, apart from the ROI method. Instead of targeting the entire face, the patches-based approach was used to divide the face into rectangular areas of 40 × 40 pixels. The BVP signal was then extracted individually for each of these patches. Finally, a single BVP signal was derived by averaging the per-patch signals.

#### Pipeline 3: Holistic ROI selection and frequency-domain HR extraction

A potential problem with peak-based pulse extraction from the rPPG-derived BVP signal is that, in contrast to the sharp peaks in an ECG signal, the smooth waveform of the BVP signal hampers peak detection. Moreover, BVP signal amplitude and baseline are sensitive to respiration, body movement, and changes in illumination (Hayano et al., 2005). An alternative is to analyze the BVP signal in the frequency domain.

Therefore, Pipelines 3 and 4 were implemented to test whether frequency-domain analysis could meaningfully improve rPPG accuracy. To this end, we chose a recently proposed post-processing framework based on pulse frequency demodulation (HRVCam; Pai et al., 2021). Our implementation of the HRVCam framework closely follows the steps described in Pai et al. (2021).

In the HRVCam framework, the extracted BVP signal is divided into smaller time windows.^1^ For each time window, an estimate of the signal-to-noise ratio (SNR) is obtained based on the power at the center frequency (i.e., the peak of the power spectral density [PSD]) relative to the power in its sidebands. The center frequency usually corresponds to the average HR, while the frequencies around the center frequency contain HRV information. Using this SNR estimate, optimal bandpass filter parameters for the window are selected. This maximizes the amount of HRV information retained while minimizing noise. From the filtered signal, the instantaneous frequency is extracted with the discrete energy separation algorithm (DESA-1; Maragos et al., 1993). Finally, the windowed instantaneous frequency is stitched back together to the original video length using overlap-add (W. Wang et al., 2017) and converted from hertz (Hz) to beats per minute (BPM).

In Pipeline 3, we implemented the HRVCam framework with the holistic ROI approach.

#### Pipeline 4: Patches ROI selection and frequency-domain HR extraction

In their original implementation of the HRVCam framework, Pai et al. (2021) used a modified version of the patches ROI approach. Specifically, they implemented signal weighting using the maximum ratio combining algorithm (MRC; Kumar et al., 2015). The MRC estimates a goodness metric per facial region by comparing the power at the center frequency of the recording to the power in the frequency band from 0.5 to 5 Hz. This goodness metric is then applied as a weight for each patch when computing the weighted average of the signal.

We tested in Pipeline 4 whether addition of the MRC algorithm yielded improved results beyond the benefits of moving-window bandpass filter and frequency-domain HR extraction.

A summary of the four rPPG pipelines tested in the current study is shown in Table 2.

**Table 2:** rPPG pipeline configurations included in the benchmark

	Pipeline 1	Pipeline 2	Pipeline 3	Pipeline 4
ROI selection	Holistic	Patches	Holistic	Patches
BVP extraction	POS	POS	POS	POS
Post-processing	MA filter + BP filter	MA filter + BP filter	Moving-window BP filter	Moving-window BP filter
Signal averaging	–	Simple average	–	Weighted average (MRC)
Pulse rate estimation	Peak-based	Peak-based	DESA-1	DESA-1

*POS* plane-orthogonal-to-the-skin algorithm, *MA* moving average, *BP* bandpass, *MRC* maximum ratio combining algorithm, *DESA-1* discrete energy separation algorithm

## ECG as the benchmark ground truth

### ECG preprocessing and instantaneous HR calculation

The ECG ground truth data were processed using the Python toolbox NeuroKit2 (Makowski et al., 2021). Raw ECG recordings were high-pass-filtered above 0.5 Hz using a fifth-order Butterworth filter, followed by a powerline filter at 50 Hz. R-peaks were extracted via the default NeuroKit2 peak detector, which detects QRS complexes based on the steepness of the absolute gradient of the ECG signal and subsequently identifies R-peaks based on local maxima in the QRS complex (Brammer, 2020).

All cleaned ECG signals and extracted R-peaks were visually inspected by an independent reviewer. On the basis of this review, 20 participants were excluded due to overall poor ECG signal quality. Across the remaining 77 participants, another 51 video recordings were excluded due to poor ECG signal quality. Within the retained 2,490 video recordings, 865 noisy segments (i.e., multi-second intervals) were identified. These noisy segments were interpolated based on the average inter-beat interval (IBI) of the three R-peaks preceding the gap, provided that the gap length did not exceed 3 s (or otherwise the record was excluded). Noisy segments at the start or end of a video recording were always excluded. A final ECG dataset of 2,436 video recordings was retained.

To be able to compare the ground truth ECG signal to the rPPG signal, the IBIs between consecutive R-peaks were converted to instantaneous HRs and interpolated to the original signal length of the ECG recording, using monotone cubic splines, which preserves smoothness and avoids overshoot (Fritsch & Butland, 1984). Finally, the instantaneous HR time series were downsampled to the 30 Hz sampling frequency of the rPPG signal.

### Temporal alignment of rPPG and ECG signal

When benchmarking the rPPG signal against ECG, multiple signal latencies need to be accounted for.

The ECG signal reflects the electrical activity of the heart, with the ECG peaks corresponding to the depolarization of the ventricles. In contrast, the rPPG signal reflects the blood movement in the vessels, with the rPPG peaks indicating the change from the anacrotic phase (primarily concerned with systole) to the catacrotic phase (primarily concerned with diastole) (Elgendi, 2012). Pulse arrival time (PAT), that is, the time it takes for blood ejected from the heart to reach different parts of the body, varies between and within individuals, due to metabolic and physiological factors (Jago & Murray, 1988). For cPPG measured on the fingertip, PATs are in the range of 200–540 ms (Rajala et al., 2017).

In addition, webcam recordings are subject to intrinsic system latency and other variable system delays which depend on the specific recording setup. For frame rates and resolution as used in the present study (25–30 fps, 1,280 × 720 pixels), these latencies can range from approximately 100 to 600 ms (Tanadi et al., 2020). Latency tests for the cameras used in the present study yielded an average delay of 201 ms, with a minimum of 91 ms and a maximum of 310 ms. There may be additional unknown delays due to internet transmission latencies when conducting an experiment online.

Using these reference ranges, we corrected the rPPG BVP signal with a backward shift of 500 ms in order to improve alignment with the ECG signal.

## Analysis

### Benchmarking of rPPG versus ECG ground truth

Five metrics were used to benchmark rPPG accuracy against the ECG ground truth: Pearson’s correlation coefficient (*r*), dynamic time warping (DTW), mean absolute error (MAE) of average HR, MAE of the standard deviation of IBIs (SDNN), and MAE of the root mean square of successive differences of IBIs (RMSSD).

DTW was included because it is particularly useful when time series differ in length or are affected by latency. In such cases, traditional measures of correlation, such as *r*, may be less accurate. This applies to rPPG, since the signal is subject to multiple unknown delays, including PAT and system latency. Recently, DTW has been used in two studies in the rPPG field and showed good alignment with other evaluation metrics (Haugg et al., 2022; Ontiveros et al., 2023).

For HRV benchmarking, SDNN and RMSSD were chosen because they have been shown to be particularly well suited for assessing HRV in ultrashort recordings of less than 1 min, like those in our dataset (Munoz et al., 2015; Pham et al., 2021). The dataset used for HRV benchmarking was limited to videos from the heartbeat counting and time counting tasks, both with fluorescent lights turned on. This resulted in a dataset of 678 records, that is, 76 participants with on average nine records per person. Videos from task 3 were excluded due to short length (16 s), because the reliability of SDNN and RMSSD has only been validated up to video lengths of 30–60 s (Munoz et al., 2015; O’Neal et al., 2016). Videos with fluorescent lights turned off were excluded, as these were targeted specifically at assessing the impact of suboptimal recording conditions (see section “Analysis of factors influencing rPPG accuracy”).

In addition, for the best-performing pipeline, limits of agreement (LoA) (Bland & Altman, 1999, 2007) were calculated between rPPG- and ECG-derived estimates of average HR, SDNN, and RMSSD.

Comprehensive descriptions of all evaluation metrics are provided in Appendix A.

### Sensitivity of rPPG for detecting group-level differences in HRV

In the next step, we assessed whether the rPPG-derived HRV estimates were sufficiently sensitive for detecting group-level differences related to participant characteristics. Participant-level averages of SDNN and RMSSD were calculated, and for both metrics, two separate linear mixed-effects models were estimated—one using the rPPG data and one using the ECG data. Each model included age, gender, BMI, and physical activity level as predictors, and a by-participant random intercept to account for multiple recordings per participants. Model assumptions were confirmed using standard diagnostics (residual distributions, residual-versus-fitted plots, variance inflation factors). These analyses provided an initial comparison of whether rPPG and ECG agreed on which participant characteristics had significant effects on SDNN and RMSSD.

We used bootstrap equivalence testing to formally assess whether effect size estimates from the rPPG dataset fell within physiologically comparable ranges to those from the ECG dataset. To preserve the nested data structure, we used cluster bootstrapping, resampling participants with replacement and retaining all their recordings. We generated 10,000 such bootstrapped datasets. For each bootstrapped dataset and for each HRV metric (SDNN and RMSSD), two separate linear models were fitted: one predicting rPPG-derived HRV metrics and the other predicting ECG-derived HRV metrics from participant characteristics (age, gender, BMI, physical activity level). We then computed the bootstrapped distribution of slope differences between measurement methods (Δ*β* = *β*_rPPG_ − *β*_ECG_) and evaluated it against the physiologically informed smallest effect size of interest (SESOI). SESOI was set to ±15 ms for both SDNN and RMSSD, reflecting typical variability in short-term HRV metrics relative to long-duration recordings (Munoz et al., 2015), as well as typical demographic effect sizes for short-term HRV metrics (O’Neal et al., 2016; Voss et al., 2013, 2015). rPPG results were considered equivalent to the ECG ground truth when at least 90% of bootstrapped differences fell within SESOI bounds.

### Analysis of factors influencing rPPG accuracy

Despite its promise, rPPG is known to be vulnerable to a number of factors, including illumination variations and movement artifacts (de Haan & Jeanne, 2013; Haugg et al., 2022; Z. Yang et al., 2022a, 2022b). Both are likely to be issues in online research studies, where participants complete experiments in their home environment. An additional concern is that rPPG accuracy may be lower for darker skin tones because higher melanin content leads to greater light absorption and lower SNR (Nowara et al., 2020). Notably, this challenge is not unique to rPPG and similarly affects cPPG (Dasari et al., 2021; Fine et al., 2021).

To assess whether recording conditions or personal characteristics might affect rPPG performance, we coded each video for video length, illumination, skin tone, head movement/rotation, and the presence of objects covering the face (e.g., glasses, facial hair, long fringes, and accessories). Details on the coding procedure can be found in Appendix D.

A linear mixed model was then fit using the R package AFEX (Singmann et al., 2024) to assess whether any of these factors impacted rPPG accuracy (as measured by DTW distance scores). By-participant random intercepts and by-participant random slopes for video length, total movement, and illumination were included in the model specification. Prior to model fitting, model assumptions were confirmed usings standard diagnostics, and model fit was confirmed using the R package DHARMa (version 0.4.6 Hartig, 2022). All variables were prepared to optimally fulfill model requirements. The dependent variable, DTW distance, was log-transformed due to pronounced positive skew (see Appendix E for a histogram). Model results are therefore reported with exponentiated coefficients and interpreted as multiplicative effects on DTW distances. Continuous variables were standardized, and variables without a meaningful zero point (i.e., video length, Fitzpatrick type, and luminance) were mean-centered. No interaction terms were modeled.

Model fitting was performed iteratively, starting with the most complex model specification including correlations among random-effects slopes and all higher-order interactions. This model resulted in a singular fit, with near-perfect correlations between random-effects terms (|*r| > *.95) and zero-variance estimates for several random slopes, indicating overparameterization. The random-effects structure was therefore simplified stepwise by first removing correlations among random slopes and then dropping unsupported interaction terms; random slopes for video length and luminance were additionally removed due to minimal between-participant variance. Finally, due to very high multicollinearity and unstable estimates for three-way fixed-effects interactions, only two-way interactions were retained in the final model. The resulting model converged successfully and yielded stable parameter estimates.

As a robustness check, we refit the maximal model using glmmTMB (Brooks et al., 2017), testing multiple optimizers. Although the maximal converged with the L-BFGS-B [limited-memory Broyden–Fletcher–Goldfarb–Shanno algorithm for handling bound constraints] optimizer, it showed the same diagnostic issues (degenerate random-effects terms and unstable three-way interactions). By contrast, the parsimonious model provided a substantially better fit (ΔAIC [Akaike information criterion] = −215) and highly similar fixed-effects estimates. Consistent with best-practice recommendations for linear mixed-effects modeling (Matuschek et al., 2017), we therefore retained the parsimonious model.

### Analysis of the emotion anticipation task

The behavioral paradigm was analyzed independently for both the rPPG and the ECG data, using linear mixed models. For both datasets, the same model specification and model fitting processes were employed. Effect sizes were compared between the two datasets using bootstrap equivalence testing.

To prepare the data for analysis, instantaneous HRs were converted to change scores relative to a 1-s baseline before trial start. Change scores were binned across time by resampling them every half-second (Poli et al., 2007).

In previous research, a triphasic cardiac response to the anticipation of emotional stimuli was observed (Poli et al., 2007): specifically, a brief initial HR deceleration (deceleration 1 [D1]) during display of the word (stimulus 1) announcing the emotional content of the picture (stimulus 2), followed by a slight HR increase (acceleration 1 [A1]) and then continued deceleration (deceleration 2 [D2]), with further deceleration when the picture was displayed on screen (picture deceleration period [PDP]). Following this analytical approach, the same components were adopted for the current study: D1 (−6,000 to −4,000 ms before S2), A1 (−4,000 to −2,000 ms before S2), D2 (−2,000 to 0 ms before S2), and PDP (0 ms to 2,000 ms after onset of S2).

The effect of component (D1, A1, D2, PDP) and stimulus condition (“Object,” “Injury,” “Erotica”) on HR change scores was assessed by fitting linear mixed models to both datasets. Prior to model fitting, orthogonal contrast codes for the condition and stimulus predictors were defined. In addition, to place both models on a common reference frame, HR change values from both datasets were *z*-standardized using the mean and standard deviation of the ECG datasets, allowing model coefficients to be interpreted as standardized effect sizes. Model fitting was performed iteratively, starting with the maximal random-effects structure, including by-participant random intercepts and by-participant random slopes for component and condition, as well as their interaction. Models were initially fit using AFEX (Singmann et al., 2024), and when convergence issues occurred were refit using glmmTMB (Brooks et al., 2017), which provides more robust optimizers for complex random-effects structures. Statistical assumptions were confirmed using the R software package DHARMa (Hartig, 2022). The maximal model failed to converge across estimation engines and optimizers (bobyqa, nlminb, BFGS, and L-BFGS-B), due to very high correlations between multiple random-effects terms (|*r|* > 0.9). Following recommendations for handling overparameterized models (Matuschek et al., 2017; Singmann & Kellen, 2019), we refit the model without correlations between random-effects terms. This reduced model yielded a boundary singular fit with lmer but successfully converged with glmmTMB using the default optimizer. Fixed-effects estimates were identical across afex and glmmTMB. We therefore accepted the converged model as the final model. The final model specification was identical for the rPPG and ECG datasets.

To formally assess whether rPPG-derived model estimates were statistically and practically comparable to those derived from the ECG ground truth dataset, we conducted an equivalence test using bootstrapping. A total of 10,000 bootstrapped samples with replacement were generated, and a generalized linear mixed model was fitted to each dataset, with HR change scores as the outcome, fixed effects for component, condition, and their interaction, and by-participant random intercepts and random slopes for component, condition, and their interaction. For each fixed-effects term, we computed the distribution of standardized differences between rPPG and ECG model estimates by subtracting the bootstrapped ECG estimate from the corresponding rPPG estimate on every iteration. Equivalence was defined as ≥ 90% of differences falling within a predefined SESOI of standardized effect sizes (Lakens, 2017). We evaluated a conservative SESOI of ±0.1 *SD*, as well as more liberal SESOI of ±0.2 *SD*, consistent with thresholds adopted in previous benchmark studies of cPPG for HRV measurement (Burma et al., 2024; Correia et al., 2020; Holmes et al., 2020).

## Results

### Benchmark results for rPPG versus ECG ground truth

We benchmarked four rPPG pipelines against ECG-derived instantaneous HR trajectories, using five predefined accuracy metrics (correlation, DTW, |∆HR|, |∆SDNN|, |∆RMSSD|). Summary statistics across all valid recordings are presented in Table 3.

**Table 3:** Evaluation metric results for the different rPPG pipelines tested in the current study

		*r*	DTW	|∆ BPM|	|∆ SDNN|	|∆ RMSSD|
**Pipeline 1** (holistic; time-domain)	Mean	.39	2.74	2.09	24.25	45.64
	*SD*	.24	2.30	3.19	21.87	27.33
	*MAD*	.20	0.96	0.58	9.18	12.29
**Pipeline 2** (patches; time-domain)	Mean	.37	3.08	2.71	26.58	48.58
	*SD*	.25	2.74	4.15	25.82	32.51
	*MAD*	.20	1.11	0.65	10.13	13.27
**Pipeline 3** (holistic; frequency-domain)	Mean	.56	1.55	1.67	11.45	11.02
	*SD*	.26	4.15	2.64	14.34	9.78
	*MAD*	.19	0.33	0.48	4.75	5.44
**Pipeline 4** (patches; frequency-domain)	Mean	.37	2.05	2.66	15.46	12.77
	*SD*	.25	3.12	3.63	17.64	10.43
	*MAD*	.19	0.45	0.74	6.70	5.99

*MAD* median absolute deviation, *DTW* normalized dynamic time warping distance, *|∆ BPM|* average absolute HR difference, *|∆ SDNN|* average absolute difference of the standard deviation of normal-to-normal inter-beat intervals, *|∆ RMSSD|* average absolute difference of the root mean square of successive differences. All values were rounded to two decimal points for better readability

**Fig. 1:**
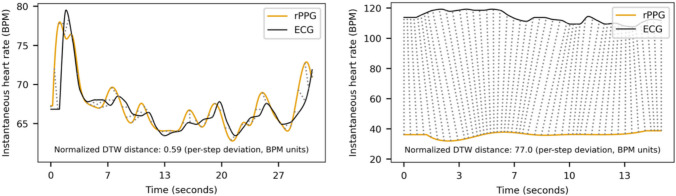
Dynamic time warping path for two example recordings. Dotted lines indicate Euclidean distances between aligned samples (based on optimal warping path). Left: recording with the smallest DTW distance between rPPG and ECG. Right: recording with the largest DTW distance. Normalized DTW reflects the average alignment cost between rPPG- and ECG-derived instantaneous HR trajectories, accounting for both differences in BPM values and differences in timing along the warping path (lower values indicate closer correspondence)

Across all metrics, the best results were obtained with Pipeline 3. This pipeline implements the POS algorithm with a holistic ROI approach and employs pulse frequency demodulation for instantaneous HR extraction. It yielded the strongest correlations between rPPG- and ECG-derived instantaneous HR time series on average across all video recordings (mean *r = *.56), as well as the maximum single-recording correlation (*r = *.97). DTW distances supported these results, with Pipeline 3 showing the lowest normalized per-step DTW distance (mean = 1.55) and tight alignment for most recordings (MAD = 0.33), despite a small number of poorly aligned outliers (*SD* = 4.15; see Fig. 1 for an illustrative example and Appendix E for the full distribution of DTW distances). Recording-level summary measures for HR and HRV metrics exhibited the same pattern, with Pipeline 3 performing best. Across recordings, HR deviated on average by only 1.67 BPM (*SD* = 2.64 BPM, *MAD* = 0.48 BPM) from the ground truth; HRV deviations were 11.45 ms for SDNN (*SD* = 14.34 ms, *MAD* = 4.75 ms) and 11.02 ms for RMSSD (*SD = *9.78 ms, *MAD* = 5.44 ms). These values further improved when recording-level estimates were aggregated at the participant level (|*Δ* BPM|_mean_ = 1.20 ± 1.28 BPM, |*Δ* SDNN| _mean_ = 8.83 ± 10.88 ms, |*Δ* RMSSD| _mean_ = 9.02 ± 7.63 ms).

**Fig 2:**
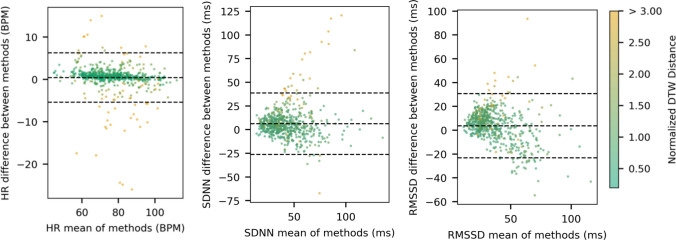
Bland–Altman plots for the best-performing rPPG pipeline (i.e., Pipeline 3) versus ECG ground truth. Dotted lines indicate limits of agreement (i.e., the range within which 95% of future differences are expected to fall). Left: average HR per recording. Middle: SDNN per recording. Right: RMSSD per recording

Limits-of-agreement analysis (LoA; Bland & Altman, 1999, 2007) further confirmed close correspondence between HR and HRV metrics across rPPG Pipeline 3 and ECG (Fig. 2). On average, rPPG slightly overestimated HR by 0.45 BPM, with 95% of future differences expected to fall between −5.39 and 6.28 BPM. Similarly, HRV metrics from rPPG showed little bias (SDNN, 6.31 ms; RMSSD, 3.68 ms), though with wider LoA ranges (SDNN, −27.10 to 39.71 ms; RMSSD, −23.65 to 31.00 ms), indicating greater uncertainty in HRV estimates than in HR estimates.

Given its consistently superior performance, only Pipeline 3 was retained for subsequent analyses.

### Sensitivity of rPPG for detecting group differences in HRV

To assess whether rPPG is precise enough to detect meaningful group differences, we examined how HRV varied with participant characteristics, and whether rPPG and ECG yielded comparable estimates of any such group differences.

In the first step of the analysis, we fit separate mixed-effects models to rPPG and ECG data to identify which of the measured participant characteristics (age, gender, BMI, physical activity level) influenced HRV metrics. For SDNN, none of the predictors showed significant effects in either measurement method. For RMSSD, female participants exhibited on average 11.2 ms higher RMSSD than male participants (*β* = 11.2 ms, 95% CI [1.7, 20.7], *p* =.021) when measured with ECG, whereas this effect was absent in the rPPG dataset (*β* = 1.39 ms, 95% CI [−5.98, 8.76], *p* =.707). No other statistically significant effects were observed (see Appendix F, Table 1).

In the second step of the analysis, we employed bootstrap equivalence testing (*n* = 10,000) to formally assess whether the magnitude of any observed differences between rPPG and ECG were physiologically relevant. For each bootstrapped sample, fixed-effects estimates were obtained separately for rPPG and ECG, and the distribution of their differences was compared to a physiologically informed SESOI of 15 ms. Across all predictors, more than 95% of differences between rPPG and ECG fell within this SESOI (see Appendix F, Table 2), indicating negligible differences between measurement methods. This included the female-male difference in RMSSD (mean Δ*β* = –9.75 ms, 95% CI [−15.09, −4.59], 97.3% within SESOI bounds).

### Factors influencing rPPG accuracy

Next, we assessed whether rPPG performance in our dataset was impacted by any of the known rPPG vulnerabilities such as excessive participant movement or suboptimal illumination. Table 4 summarizes the results of the linear mixed model assessing the influence of recording conditions and personal characteristics on rPPG accuracy (measured by DTW distance, where smaller distance indicates greater accuracy). The model explained 20.2% of the variance in DTW distance by fixed effects alone (marginal *R*^2^ =.202) and 65.9% when accounting for both fixed and random effects (conditional *R*^2^ =.659).

**Table 4:** Linear mixed model results assessing the relationship between rPPG accuracy (measured by DTW distance) and recording conditions/individual differences

Fixed effects						
	log(*B*)	exp(*B*)	95% CI	*SE* (log)	*t*	*p*
Intercept	−0.35	0.71	0.64 to 0.78	0.05	−6.64	<.001***
Video length	−0.09	0.91	0.87 to 0.96	0.03	−3.39	<.001***
Total movement	0.28	1.33	1.2 to 1.46	0.05	5.52	<.001***
Proportion of frames hands visible	0.29	1.33	1.07 to 1.65	0.11	2.62	.029*
Skin type (Fitzpatrick)	0.16	1.17	1.06 to 1.3	0.05	3.02	.003**
Luminance	−0.15	0.86	0.79 to 0.93	0.04	−3.73	<.001***
Skin type × luminance	−0.10	0.91	0.84 to 0.98	0.04	−2.57	.012*

Smaller DTW distance indicates greater accuracy. The model included video length, participant movement, the proportion of video frames in which hands were visible, skin type (Fitzpatrick scale), illumination, and whether any items covered the participant’s face. The dependent variable was log-transformed due to skewed distribution. log(B) = unstandardized coefficient. exp(B) = exponentiated coefficient (multiplicative effect on Y). Significance levels: *** *p* <.001, ** *p* <.01, * *p* <.05. Only significant fixed effects are shown here. For the full results table, see Appendix G

**Fig 3:**
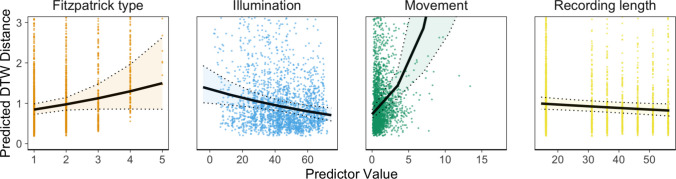
Factors affecting rPPG accuracy visualized based on marginalized fixed-effects predictions. From left to right: DTW distance (smaller distance indicates greater accuracy) as a function of Fitzpatrick type (1 = very fair to 6 = dark brown), illumination strength (luminance value of LAB color spectrum), subject movement (standardized units), and video length (seconds). Solid lines indicate marginalized fixed-effects predictions from the linear mixed model. Dotted lines indicate 95% confidence intervals. Points represent scores for individual recordings in the original dataset. The *y*-axis was trimmed at a DTW distance of 3 for readability.

Results indicated that rPPG accuracy was significantly lower for videos with greater participant movement, exp(*β*) = 1.33, 95% CI [1.07, 1.65], *p* <.001, and when a participant’s hands were visible in the video, exp(*β*) = 1.33, 95% CI [1.20, 1.46], *p* =.029. In contrast, rPPG accuracy increased with video length, exp(*β*) = 0.91, 95% CI [0.87, 0.96], *p* <.001, and higher illumination level, exp(*β*) = 0.86, 95% CI [0.79, 0.93], *p* <.001. Darker skin tones (higher Fitzpatrick types), exp(*β*) = 1.17, 95% CI [1.06, 1.30], *p* <.001, were associated with lower rPPG accuracy (Fig. 3).

**Fig 4:**
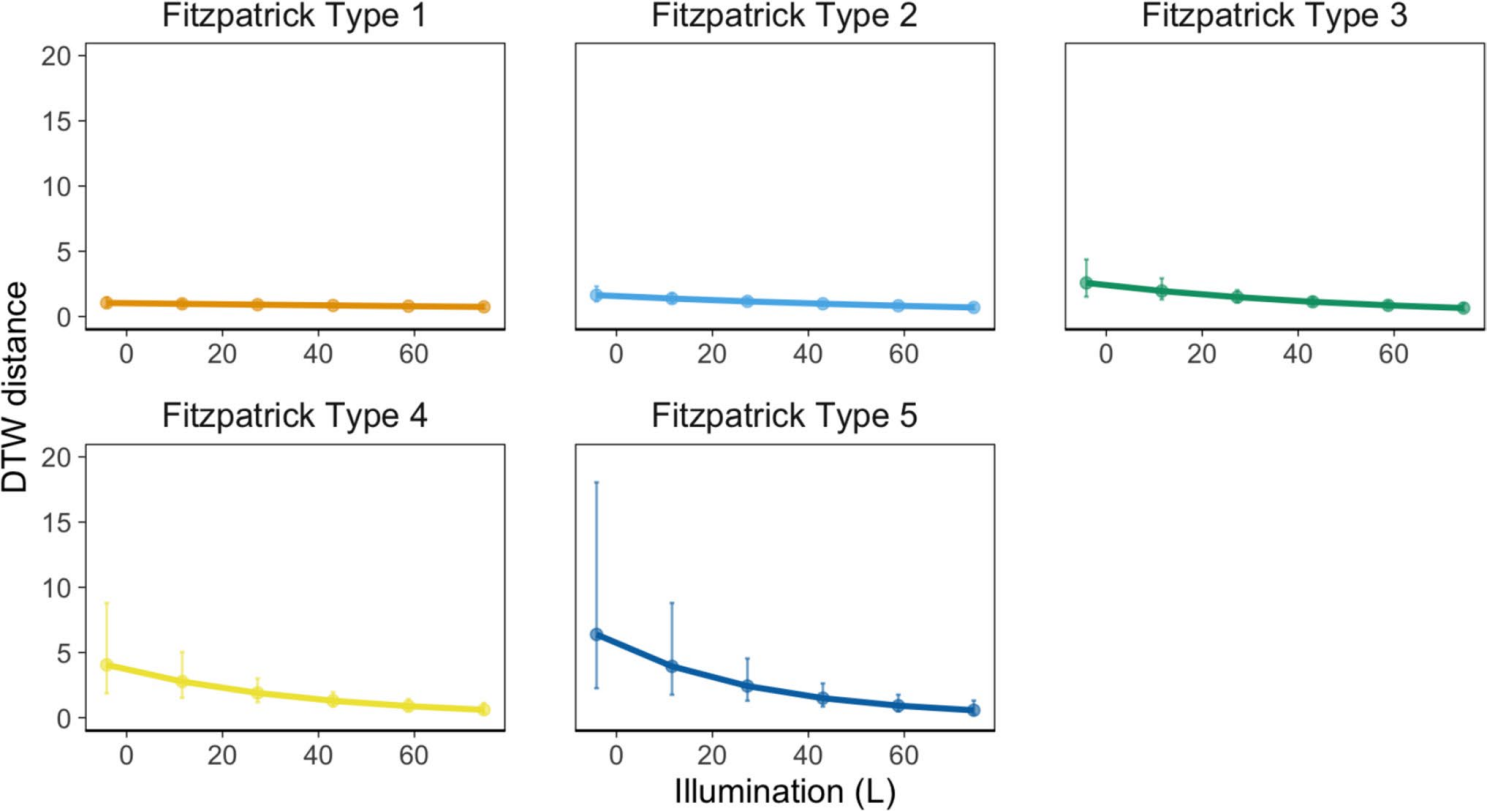
The effect of illumination level on rPPG accuracy by Fitzpatrick type. Error bars indicate 95% confidence intervals. Illumination was measured with the luminance (L) component of the LAB color spectrum. No plot is shown for Fitzpatrick type 6, as there were no participants in this group.

Additionally, a significant interaction was observed between Fitzpatrick skin type and illumination level, exp(*β*) = 0.91, 95% CI [0.84, 0.98], *p* =.012, suggesting that the effect of illumination differed by skin type. Inspection of the marginalized fixed-effects predictions from the linear mixed model revealed that as luminance values increased, the performance differences between skin types diminished. For luminance values lower than 30, the DTW distance for Fitzpatrick type 5 was estimated to be on average 2.25 BPM higher than that for Fitzpatrick type 1. In contrast, for luminance values of 30 and above, the difference in DTW distance between Fitzpatrick type 5 and Fitzpatrick type 1 was estimated to be only 0.21 BPM (Fig. 4).

### Analysis of the emotion anticipation task

**Fig 5:**
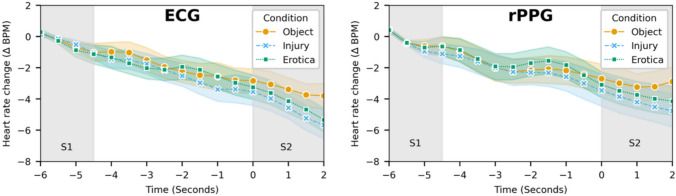
HR changes during the emotion anticipation task. Left: ECG data. Right: rPPG data. S1 indicates the time during which the word announcing the emotion category was displayed on screen. S2 indicates the time during which the corresponding picture was shown on screen. The time axis is relative to the onset of S2

For both rPPG and ECG, the emotion anticipation paradigm data showed patterns aligned with previous research: initial HR deceleration, followed by a brief period of HR recovery, and then further deceleration (Fig. 5). HRs for the object category started to recover once the picture appeared on screen, while for emotional pictures (injury and erotica), the deceleration continued into the time during which the picture was displayed.

For each of the two datasets—rPPG and ECG—a separate generalized linear mixed model was fit (Table 5). To place both models on a common reference scale, HR change scores were *z*-standardized using the mean and standard deviation of the ECG dataset, allowing coefficients to be interpreted as standardized effect sizes. The model fitted to the rPPG data explained 10.2% of the total variance with fixed effects alone (marginal *R*^2^ =.102), and 22.9% with fixed and random effects together (conditional *R*^2^ =.229). This was slightly less than the variance explained by the model fitted to the ECG data, which explained 13.4% with fixed effects alone (marginal *R*^2^ =.134) and 27.6% with fixed and random effects together (conditional *R*^2^ =.276). In line with this, the rPPG dataset exhibited larger residual variability (variance = 1.03, *SD* = 1.01) than the ECG dataset (variance = 0.73, *SD* = 0.85).

**Table 5:** Results for the two separate linear mixed models fitted to the emotion-anticipation task data (ECG and rPPG)

Fixed effects								
	*β*		95% CI		*z* value		*p* value	
	ECG	rPPG	ECG	rPPG	ECG	rPPG	ECG	rPPG
(Intercept)	0.00	0.02	−0.07 to 0.07	−0.05 to 0.09	0.09	0.64	.927	.523
Object vs. Erotica	−0.01	0.00	−0.06 to 0.05	−0.06 to 0.07	−0.29	0.09	.770	.930
Object vs. Injury	0.08	0.07	0.02 to 0.14	−0.01 to 0.14	2.51	1.78	.012*	.076
D1 vs. D2	0.26	0.18	0.19 to 0.33	0.10 to 0.26	7.48	4.53	<.001***	<.001***
A1 vs. D1&D2	0.62	0.74	0.56 to 0.68	0.67 to 0.80	20.49	20.90	<.001***	<.001***
All vs. PDP	0.55	0.45	0.49 to 0.60	0.38 to 0.51	19.60	13.50	<.001***	<.001***
Object vs. Injury * All vs. PDP	−0.08	−0.05	−0.16 to −0.01	−0.15 to 0.04	−2.14	−1.15	.032*	.251
Random effects								
				Variance		*SD*		
				ECG	rPPG	ECG	rPPG	
Participant:		Intercept		0.085	0.081	0.29	0.28	
		Object vs. Erotica		0.036	0.056	0.19	0.24	
		Object vs. Injury		0.049	0.077	0.22	0.28	
		D1 vs. D2		0.005	<.001	0.07	<.001	
		A1 vs. D1/D2		0.004	<.001	0.06	<.001	
		Residual		0.728	1.027	0.85	1.01	
		Observations		4980	4972			
		Marginal *R*^2^		0.134	0.102			
		Conditional *R*^2^		0.276	0.229			

Only significant interaction terms are displayed. Random-slope effects with near-zero variance are omitted. Cardiac response components: D1 = first deceleration period. D2 = second deceleration period. A1 = first acceleration period. Contrast “All vs. PDP” compares the picture-viewing period (PDP) to all other cardiac-response components. Reported *β* coefficients are *z*-standardized effect sizes based on the ECG dataset (using ECG means and standard deviations for scaling). *** *p* <.001, ** *p* <.01, * *p* <.05. Full results are available in Appendix H

Both models supported significant fixed effects for all contrasts relating to cardiac response components, with small effect sizes for contrast D1 versus D2 (*β* = 0.26 for ECG; *β* = 0.18 for rPPG) and moderate effect sizes for the other two contrasts A1 versus D1&D2 (*β* = 0.62 for ECG; *β* = 0.74 for rPPG) and All versus PDP (*β* = 0.55 for ECG; *β* = 0.45 for rPPG). One fixed effect differed between rPPG and ECG. The ECG model showed a very small interaction effect for the contrast between “Object” versus “Injury” and PDP versus all other periods (*β* = −0.08), whereas the corresponding interaction effect in the rPPG model was even smaller and nonsignificant (*β* = −0.05).

Random-effects variance structures were highly similar across the two models. Both models showed the largest between-participant variability in the intercept (ECG, *σ*^2^ = 0.085; rPPG, *σ*^2^ = 0.081), followed by random slopes for the condition contrasts (Object vs. Erotica: ECG, *σ*^2^ = 0.036; rPPG, *σ*^2^ = 0.056; Object vs. Injury: ECG, *σ*^2^ = 0.049; rPPG, *σ*^2^ = 0.077). This pattern indicates meaningful individual differences in average cardiac response as well as in reactivity to different emotion categories. Between-participant variability in cardiac response across time—captured by the component contrasts—was very small in the ECG model (*σ*^2^ = 0.004–.005) and negligible in the rPPG model (*σ*^2^ <.001).

**Fig 6:**
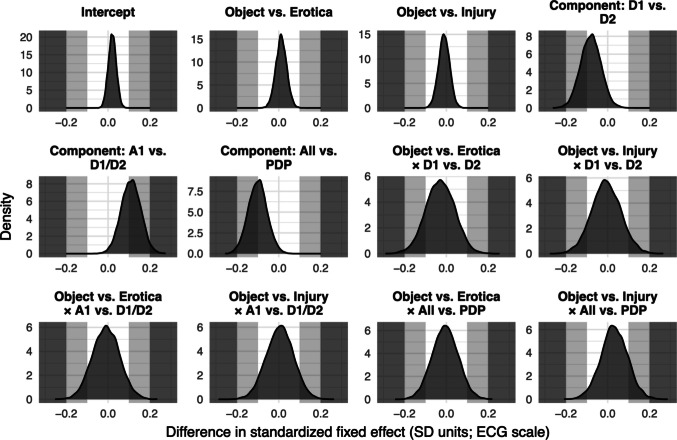
Distributions of bootstrapped standardized differences between rPPG- and ECG-derived fixed-effects estimates. Distributions centered near zero indicate good agreement between methods. Shaded bands denote predefined smallest-effect-size-of-interest (SESOI) ranges used to judge practical equivalence: < ±0.1 *SD* (white/no shading; negligible difference); ±0.1 to ±0.2 *SD* (light gray; small difference); > ±0.2 *SD* (dark gray; moderate to large difference)

Moreover, we formally assessed whether the rPPG-derived model estimates were comparable to those from the ECG ground truth using bootstrap equivalence testing. The bootstrapped distributions of standardized differences between rPPG- and ECG-derived fixed-effects estimates (Fig. 6) indicated overall close agreement between measurement methods. For all fixed-effects terms, the vast majority (≥ 90%) of bootstrapped rPPG-ECG differences fell within ±0.2 *SD*, indicating small deviations between modalities. Using the more stringent ±0.1 *SD* threshold, strong agreement was observed for model intercepts and the fixed-effects terms related to emotion category, while differences for component-related terms (coding for the time course of HR changes) and their interaction terms were more variable, with less than 90% within the ±0.1 *SD* threshold.

## Discussion

rPPG enables the extraction of cardiac signals from videos recorded with standard consumer-grade cameras, offering a cost-effective and accessible alternative for remote and resource-limited research settings. However, it remains unclear whether rPPG can accurately capture moment-to-moment fluctuations in cardiac activity and provide reliable estimates of HRV indices, as ecologically valid benchmarks with larger samples sizes remain scarce.

To address this gap, we collected video and ECG data from 77 participants performing three behavioral tasks under controlled laboratory conditions, designed to mirror typical at-home recoding setups. Using this dataset, we implemented the widely used POS algorithm, which has repeatedly been shown to outperform other rPPG algorithms, including machine learning methods (Haugg et al., 2022; Unakafov, 2018; van Es et al., 2023; Zhu et al., 2023), alongside a variant incorporating adaptive bandpass filter and pulse frequency demodulation in the post-processing steps (HRVCam; Pai et al., 2021). Each implementation was tested using both holistic and patch-based ROI selection, and all four configurations were benchmarked against the ECG gold standard using five predefined evaluation metrics.

The best-performing rPPG pipeline in our benchmark was Pipeline 3, implementing POS with HRVCam framework and holistic ROI selection. This configuration consistently outperformed the standard POS implementation. Average HR deviated by less than 2 BPM from the ECG gold standard, and HRV estimates deviated by less than 12 ms from the ECG gold standard (SDNN = 11.45 ± 14.34 ms, RMSSD = 11.02 ± 9.78 ms). These values are broadly comparable to those reported by Pai et al. (2021) in their initial evaluation of the HRVCam framework in a smaller sample of 16 participants. In their study, estimation errors were lowest during low-movement conditions such as reading and video watching (SDNN: 3 and 2 ms MAE; RMSSD = 4 and 5 ms MAE), and substantially higher when participants were talking (SDNN = 9 ms; RMSSD = 14 ms).

We observed a moderate correlation between the pipeline’s instantaneous HR trajectories and those derived from the ECG ground truth (*r* =.56). This is not unexpected, as correlation is highly sensitive to even small timing discrepancies, while achieving exact temporal alignment between rPPG and ECG signal is challenging due to distinct waveforms, intrinsic system latencies, and physiological delays between BVP and ECG signals. While ECG remains the gold standard for HRV measurement, future work could benchmark the HRVCam framework or other HRV-focused rPPG implementations against cPPG, as this would facilitate temporal alignment between the compared signals, given similar waveforms. For our study, DTW distance provides a more meaningful metric than correlation, as it explicitly accommodates timing offsets between compared signals (Müller, 2007). We observed an average normalized per-step DTW distance of only 1.55 (i.e. BPM-consistent units, reflecting both differences in absolute HR and temporal shifts along the warping path), which is lower than values previously reported in an rPPG benchmark (Haugg et al., 2022).

Together, these findings highlight the benefit of dedicated rPPG implementations for HRV extraction. The pulse-frequency demodulation, employed for post-processing in Pipelines 3 and 4, is better suited to the smooth waveform of the rPPG signal (Hayano et al., 2005), and the adaptive, physiologically informed bandpass filter constrains instantaneous HR estimates to realistic ranges and stabilizes them relative to preceding signal windows (Pai et al., 2021).

It is surprising that both tested pipelines using holistic ROI selection outperformed their counterparts with patch-based ROIs. Although the holistic approach is the most widely used approach in rPPG research, prior work generally reports better accuracy when multiple patches are combined (Bondarenko et al., 2025). This is attributed to systematic differences across facial regions in light reflectance, skin thickness, blood perfusion, and susceptibility to facial-expression-related motion, with the forehead and cheeks usually yielding best results (Boccignone et al., 2025; D.-Y. Kim et al., 2021; Kwon et al., 2015). Moreover, patch-based implementations that incorporate SNR-based weighting, as in Pipeline 4 in our study, are generally regarded as superior, because signal weighting downweighs low-quality regions and guards against performance deterioration due to sudden changes in illumination that affect only parts of the face (Bondarenko et al., 2025; Kiddle et al., 2023; Kumar et al., 2015). However, holistic ROI selection may match or even outperform patch-based methods under steady, uniform illumination—where reflectance variability is minimal—and when patches become occluded or drift onto the background during large head rotation (Boccignone et al., 2022; Haugg et al., 2022; Nagar et al., 2024). Although we employed two lighting conditions (fluorescent lights on/off), we did not vary illumination within videos, and participants were explicitly instructed to limit head movement. Furthermore, our patch-based pipelines employed a single configuration, limiting conclusions about optimal patch design. Future research should systematically examine these factors, particularly in the context of HRV measurement.

An important consideration for the real-world application of rPPG, for example in online experiments, is whether precision is sufficient to detect group differences. We therefore assessed whether HRV metrics in our sample differed in relation to age, gender, BMI, or physical activity level, and whether rPPG and ECG yielded comparable estimates of these effects. Our results showed very limited group differences in HRV overall, likely owing to the relatively homogeneous demographics of our sample, which predominantly comprised young, healthy, adults. The exception was a gender effect for RMSSD, with female participants on average exhibiting 11.2 ms higher values than males when measured with ECG, but not with rPPG. This finding is surprising, since—while limited—previous studies of short-term HRV indices did find statistically significant age-related differences in both SDNN and RMSSD but no sex/gender effects (O’Neal et al., 2016; Voss et al., 2015). It should also be noted that the observed effect size is close to the known measurement error of ultrashort-term HRV metrics, relative to standard 5-min short-term HRV measurements (Munoz et al., 2015), making it unclear whether this observation represents a genuine physiological effect or random sampling variation. Larger short-term HRV effects are observed, for example, for postural manipulations (e.g. supine vs. standing) (Besson et al., 2025), and future benchmark of rPPG-derived HRV metrics could leverage such manipulations.

We further tested the performance of rPPG in a typical behavioral experiment, where participants completed an emotion anticipation task (Poli et al., 2007). In both datasets, a typical triphasic cardiac response was observed, with highly significant results and substantial between-participant variability in average response. The key difference between measurement methods was that the model fit to the ECG data identified a significant interaction between the contrast comparing the “Object” and “Injury” conditions and the contrast comparing the PDP period to all other periods, which could not be confirmed with the rPPG dataset. However, the corresponding effect size in the ECG model was negligible, indicating limited practical relevance. Overall, slightly larger unexplained variance suggests that rPPG yields noisier estimates, which should be taken into account during study design. When moderate to large effects are studied in sufficiently large samples, the present data suggests that rPPG yields HRV results equivalent to those obtained from traditional contact-based ECG.

Our analysis of environmental and personal influences on rPPG accuracy confirms and extends insights from previous studies. Participant movement (including hands close to/in front of the face) and poor illumination of the participant’s face had a significant negative influence on the quality of the extracted BVP signal, whereas our pipeline performed better on longer recordings. There were also significant differences in performance by skin tone, with lower rPPG accuracy observed for darker skin (i.e., a higher Fitzpatrick type). Analysis of the interaction between Fitzpatrick type and illumination strength revealed that for videos with moderate to high illumination, the accuracy differences between Fitzpatrick types reduced significantly. Future studies should further validate this finding and, if confirmed, rPPG implementations could leverage it to automatically reject videos with illumination below a given threshold and thereby ensure comparable accuracy across different skin types.

A key limitation of our study is the use of short-duration analysis windows in all tested paradigms (16–56 s). This constrained our analysis to time-domain HRV metrics, which have been shown to be accurate for ultrashort-term recordings of less than 1 min (Munoz et al., 2015; O’Neal et al., 2016). Moreover, cardiac responses to stimuli with emotional valence exhibit substantial inter- and intra-individual differences in the timing and magnitude of anticipatory deceleration and post-stimulus rebound, which can extend beyond the trial window (Alam et al., 2023; Mezzacappa et al., 2001). Short-duration windows, although widely used in experimental psychology, may not fully capture these dynamics and may introduce measurement noise. Future work could expand the benchmark of rPPG to longer-duration recordings that allow for the modeling of more complex individual cardiac response trajectories and permit the estimation of frequency-domain HRV metrics.

## Conclusion

In summary, our study confirmed that rPPG is a promising technology for remote collection of cardiac data, with potential applications in research and industry. Recent improvements in rPPG algorithms and pre- and post-processing pipelines have contributed to making the method more robust. The current level of accuracy appears sufficient to measure cardiac response patterns and HRV metrics such as SDNN and RMSSD in carefully curated, high-powered studies with large sample sizes that assess cardiac parameters at the group level. At the individual level, however, only average HR measurements from rPPG should be trusted, with longer recordings yielding more accurate measurements.

For any researcher interested in the method, it is important to be aware of the vulnerabilities of rPPG algorithms. Suboptimal recording conditions, such as poor illumination and excessive participant movement, can lead to significant reductions in accuracy. Moreover, to avoid potential discrimination, performance differences for darker skin types should not be overlooked and need to be carefully mitigated, such as through optimal lighting conditions.

## Supplementary Information

Below is the link to the electronic supplementary material.

Supplementary file1 (DOCX 356 KB)

## Data Availability

The anonymized datasets generated and analyzed during the current study are available in the accompanying OSF repository (https://osf.io/nat72/?view_only=df38fe1b074547f0a48351c7a78a49cd). Due to data protection rules, the raw video recordings collected in the validation study cannot be made available.

## Footnotes

1 Pai et al. (2021) used a window length of 10 s, with a variable step size of 1–5 s, depending on the amount of motion in the video. Given the relatively short length of recordings in our dataset (ranging from 16 to 56 s), we opted for a window length of 5 s and a step size of 2.5 s. Spot checks confirmed that the performance was not degraded by the shorter window length. Future studies may test the optimal window length.

## Code availability

Stimuli, data, and analysis code are available at OSF (https://osf.io/nat72/?view_only=df38fe1b074547f0a48351c7a78a49cd). None of the reported studies were preregistered. Due to data protection rules, the raw video recordings collected in the validation study cannot be made available.

## Declarations

### Open practices statement

Stimuli, data, and analysis code are available at OSF (https://osf.io/nat72/?view_only=df38fe1b074547f0a48351c7a78a49cd). None of the reported studies were preregistered. Due to data protection rules, the raw video recordings collected in the validation study cannot be made available.
