# Supplementary material for: Advancing remote photoplethysmography (rPPG) to facilitate cardiac monitoring in naturalistic settings using webcam technology
Source: Behav Res Methods. 2026 Apr 23;58(5):135. doi: 10.3758/s13428-026-02953-x (PMC13106271; doi:10.3758/s13428-026-02953-x)
Supplement: Supplementary file 1 — Supplementary file1 (DOCX 356 KB) [file 13428_2026_2953_MOESM1_ESM.docx]

## Appendix A – Analysis of characteristics of participants excluded due to poor ECG

|  | Good ECG  (M ± SD) | Poor ECG  (M ± SD) | *t* | df | *p* | Test |
| --- | --- | --- | --- | --- | --- | --- |
| Number of participants | 77 | 20 | - | - | - | - |
| Age | 23.83 ± 3.80 | 24.60 ± 4.64 | -0.68 | 26.0 | .500 | t-test |
| Weight (kg) | 69.44 ± 15.85 | 61.01 ± 10.21 | 2.90 | 45.7 | .006 | t-test |
| Height (cm) | 170.88 ± 9.67 | 167.70 ± 9.14 | 1.37 | 31.0 | .18 | t-test |
| BMI | 23.63 ± 4.10 | 21.60 ± 2.62 | 2.72 | 46.0 | .009 | t-test |
| Sports (per week) | 2.41 ± 1.52 | 2.51 ± 1.45 | -0.27 | 30.7 | .791 | t-test |
| Fitzpatrick type  (1 light – 6 dark) | 1.69 ± 0.96 | 2.05 ± 0.94 | -1.52 | 30.1 | .139 | t-test |
| Average luminance | 42.80 ± 12.05 | 47.03 ± 9.86 | -1.63 | 35.3 | .112 | t-test |
| Average movement | 14.12 ± 8.42 | 17.26 ± 7.24 | -1.67 | 33.7 | .104 | t-test |
| Female (%) | 44.2 | 10.0 | - | - | .006 | Fisher’s exact |
| Male (%) | 54.5 | 85.0 | - | - | .006 | Fisher’s exact |
| Other gender (%) | 1.32 | 5.0 | - | - | .006 | Fisher’s exact |

## Appendix B – Illustration of selected facial regions used for skin extraction in the patches-based ROI approach


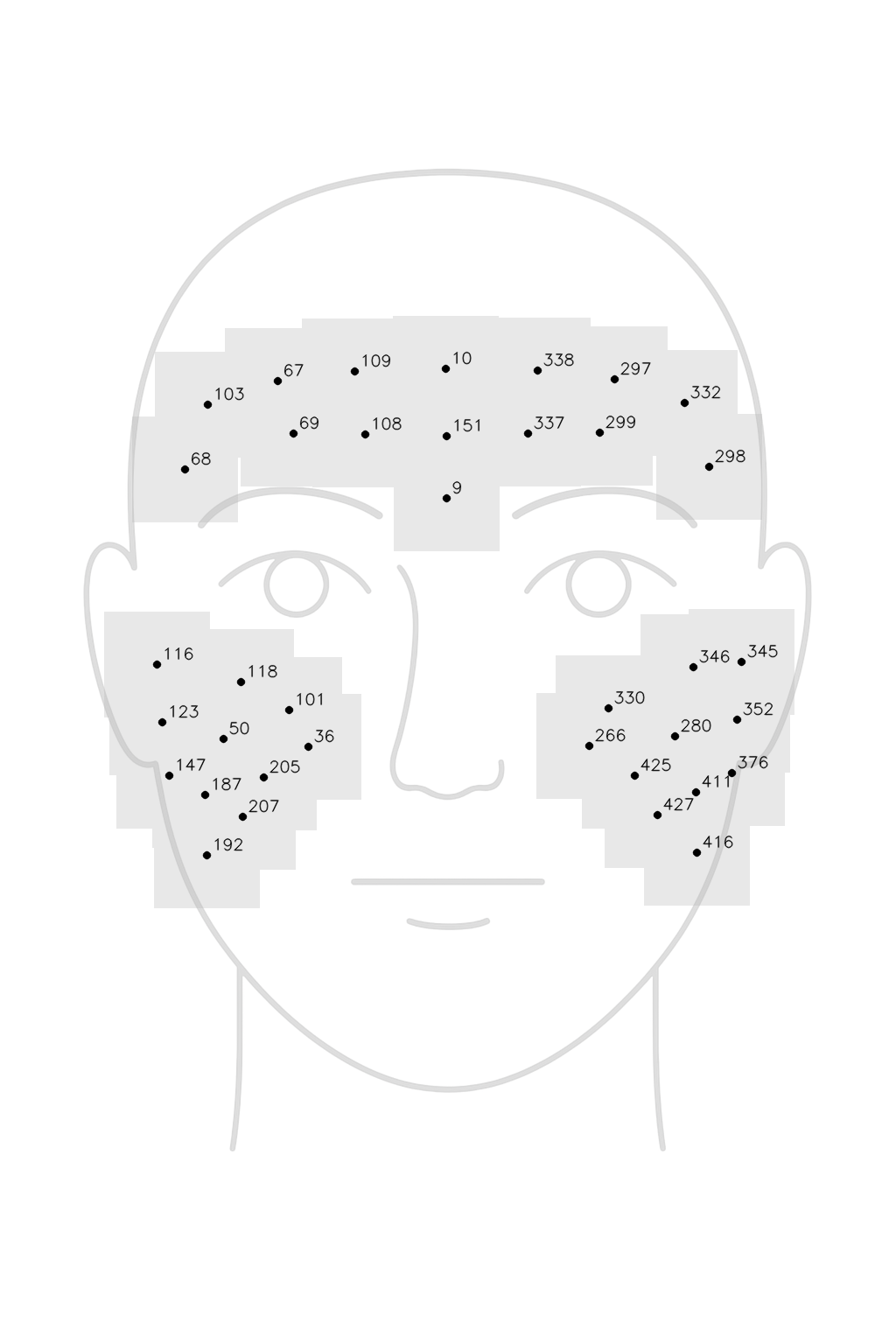


*Figure A1. Schematic illustration of the facial regions of interest (ROIs) used in the patch-based skin extraction approach. Light gray squares (40 × 40 pixels) indicate the approximate areas centered on selected MediaPipe Face Mesh (Kartynnik et al., 2019) landmarks covering the forehead and cheeks. Non-skin elements within patches were automatically excluded in subsequent steps using convex hull masking and RGB-thresholding.*

## Appendix C – Benchmark metrics

#### Pearson’s Correlation Coefficient (r)

The *r coefficient* measures the linear relationship between the rPPG and the ECG time series. *r* was calculated as follows:

$r= \frac{\sum_{i=1}^{N} (x_{i}-\bar{x})(y_{i}-\bar{y})}{\sqrt{\sum_{i=1}^{N} {(x_{i}-\bar{x})}^{2}\sum_{i=1}^{N} {(y_{i}-\bar{y})}^{2}}}$ (1)

where *x_i_* and *y_i_* denote the individual data points from the ECG and rPPG time series, respectively. *N* refers to the total number of sampling points.

#### Dynamic Time Warping (DTW)

DTW identifies the optimal alignment path between two time series by dynamically matching each data point in one time series to the closest counterpart in a defined window of the other time series. Thereafter, the dissimilarity of the two time series is quantified by calculating the remaining cumulative distance between the data points (Müller, 2007; Sakoe & Chiba, 1978). The DTW distance between two time series A and B is calculated as:

$DTW\left( A,B \right)=\min\left( \sum_{k=1}^{K} d\left( i_{k,}j_{k} \right) \right)$ (2)

where *i_k_* and *j_k_* are indices of points in A and B respectively, d(i,j) is a local dissimilarity function (such as the Euclidian distance), that measures the distance between corresponding data points, and *K* is a warping path that maps the indices of A onto the indices of B. In other words, DTW finds the optimal warping path *K* that minimizes the cumulative distance *d* between the time series. To ensure reasonable warping paths, constraints such monotonicity are imposed through defined step patterns. It is helpful to normalize the DTW distance to the average per-step distance along the warping path, particularly when comparing multiple time series of variable length. An additional benefit of DTW is that distances retain the measurement units of the input data, making them intuitive to interpret (Giorgino, 2009; Müller, 2007).

The Python package DTW (Giorgino, 2009) was used to compute the normalized Euclidian DTW distance between the rPPG and the ECG timeseries.

#### Difference in Average HR

The accuracy of rPPG algorithms can be assessed by comparing the average HR to a ground truth. This approach is often used because the pulse signal’s energy is largely concentrated in the peak of the power spectral density (PSD) of the BVP signal, making it relatively easy to detect. Since the frequency resolution of the PSD is proportional to video length, longer recordings yield more accurate HR estimates when using this approach (Wang et al., 2017).

In the current study, we calculated the absolute difference in average HR, measured in beats-per-minute (|BPM ∆|) by first determining the HR difference between rPPG and ECG at every sample point and then computing the mean of the absolute differences.

#### Difference in HRV Metrics

HRV refers to a set of metrics that index neurocardiac autonomic function and are traditionally measured from long-term ECG recordings. These metrics can roughly be categorized into three groups: time-domain, frequency-domain, and non-linear measurements.

For the measurement of low-frequency components and more complex dynamics, recordings of at least 24 hours are usually required. SDNN from 24 h recordings is regarded as the gold standard for cardiac risk stratification. High-frequency components, however, can successfully be assessed with short recordings of less than 5 minutes (Munoz et al., 2015; O’Neal et al., 2016; Pham et al., 2021; Shaffer & Ginsberg, 2017). SDNN from short-term recordings mainly reflects parasympathetically mediated respiratory sinus arrhythmia. Compared to SDNN, RMSSD is more influenced by parasympathetic nervous system activity and is therefore regarded as the primary time-domain measure of vagally-mediated changes in HRV (Shaffer & Ginsberg, 2017). For both metrics it has been shown that ultra-short measurements of 30-60 seconds can be reliable and that abnormal values are associated with increased risk of cardiovascular disease and mortality (Munoz et al., 2015; O’Neal et al., 2016). The accuracy of SDNN is likely to improve with recording duration, whereas RMSSD is particularly suited for short recordings, due to its robust statistical properties, for both (Pham et al., 2021; Shaffer & Ginsberg, 2017).

Given the short duration of the recordings in our dataset (16-56 seconds), we focused on time-based high-frequency HRV components and calculated the standard deviation of normal-to-normal intervals (SDNN) and the root mean square of successive differences (RMSSD) for all recordings with a duration of at least 30 seconds. Normal-to-normal intervals (NN intervals) refer to a heartbeat series corrected for technical and physiological artefacts (such as ectopic beats) (Pham et al., 2021). In the current study, NN intervals were extracted from IBIs by excluding IBIs shorter than 300 ms or longer than 2 seconds, IBIs that exhibited sudden jumps (i.e., a change in IBI from the previous IBI greater than 20%), and IBIs with a median absolute deviation (MAD) greater than five (Kirk et al., 2022; Lipponen & Tarvainen, 2019; Malik et al., 1996). NN intervals were then used to compute SDNN and RMSSD as follows:

$SDNN=\sigma(IBI)$ (3)

$RMSSD=\sqrt{\frac{\sum{\left( IBI\left( n \right)-IBI(n-1 \right))}^{2}}{N-1}}$ (4)

where IBI is the inter-beat interval in milliseconds, σ is the standard deviation, and *N* is the number of NN intervals in the recording.

We calculated SDNN and RMSSD separately from both rPPG and ECG and computed their absolute difference for each recording (from here on referenced as |SDNN ∆| and |RMSSD∆|, respectively).

#### Limits of agreement (LoA)

LoA (Bland & Altman, 1999, 2007) were calculated for average HR, SDNN, and RMSSD. The LoA method is widely used to compare accuracy between methods. LoA is primarily a graphical technique that plots the difference between two methods against their average, highlighting potential bias as well as the expected range of future differences. Given our experimental design, we employed the calculation method for repeated measures when the true value is not constant (Haghayegh et al., 2020).

## Appendix D – Coding of personal and video features

Skin tone and the presence of objects covering the face were coded manually by independent reviewers. Skin tone was assessed using the Fitzpatrick scale (Fitzpatrick, 1988). The Fitzpatrick scale is a 6-point scale, ranging from near-albino (1) to highly pigmented (6). It is widely used, including in the field of rPGG research (Fine et al., 2021). The presence of other objects covering the face, such as long hair, facial hair, glasses, or accessories was coded as a dichotomous variable (present/absent).

Illumination was measured as the average luminance value (L channel in the LAB color space) of all facial skin pixels. To improve the robustness of this measurement against transient illuminations variations, for each recording 10 random frames were sampled and their average luminance value was computed.

Head movement and rotation were measured with the help of the computer vision library MediaPipe FaceMesh (Kartynnik et al., 2019). Head movement was quantified by the sum of squared distances (measured in pixels) that the facial landmark corresponding to the tip of the nose (landmark index 1) moved throughout the recording. Head rotation was quantified as the sum of squared rotation angles for the same landmark, extracted using the solve_PnP function of the computer vision library OpenCV (Bradski, n.d.). Both metrics were normalized by dividing them by the video length. A total movement parameter was calculated by summing the movement and rotation values.

We also assessed whether participants moved their hands towards/in front of their face during a recording. For this, we again used the computer vision library MediaPipe Hands (Zhang et al., 2020) to measure the percentage of video frames in which hands were detected.

## Appendix E – Distribution of DTW distances


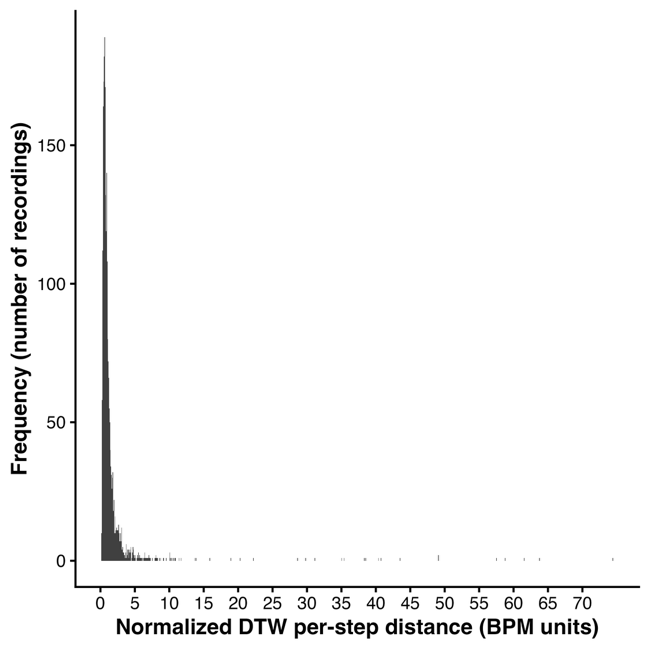

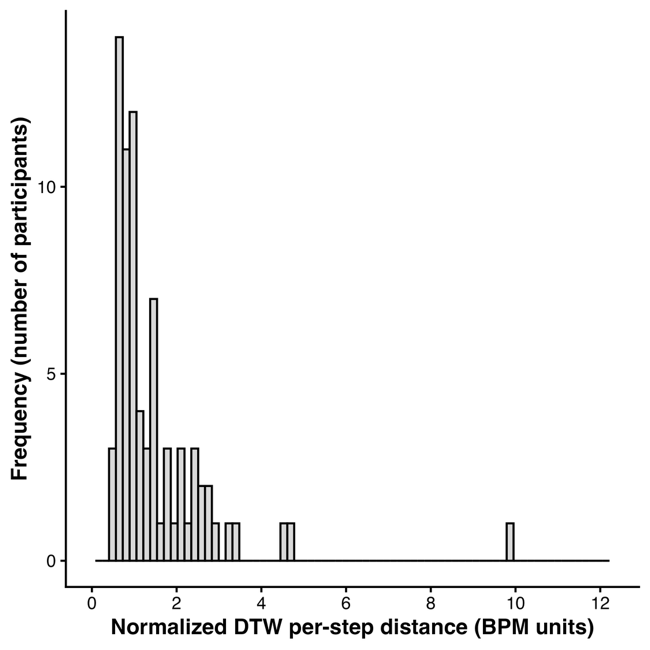


*Figure A2. Distribution of normalized dynamic time warping (DTW) distances between rPPG- and ECG-derived instantaneous HR trajectories. Left: Histogram of normalized DTW distances across all recordings. Right: Histogram of mean normalized DTW distances per participant, summarizing individual-differences in rPPG performance. The normalized DTW metric reflects the average per-step deviation between trajectories (BPM-consistent units), incorporating HR differences and DTW warping path cost (lower values indicate closer correspondence).*

## Appendix F – Results tables: Sensitivity of rPPG for detecting group differences

| Metric | Predictor | *β* ECG | *β* rPPG | ECG CI | rPPG CI | *p* ECG | *p* rPPG |
| --- | --- | --- | --- | --- | --- | --- | --- |
| SDNN | Intercept | 39.10 | 48.80 | 33.26 to 44.93 | 43.18 to 54.43 | < .001*** | < .001*** |
| SDNN | Age | −−0.55 | −0.65 | −1.58 to 0.49 | −1.65 to 0.34 | .294 | .194 |
| SDNN | Female (vs. male) | 2.45 | −3.71 | −5.74 to 10.63 | −11.58 to 4.17 | .553 | .351 |
| SDNN | Other gender | 3.35 | −0.12 | −30.89 to 37.59 | −33.29 to 33.05 | .846 | .994 |
| SDNN | BMI | −0.15 | 0.09 | −1.13 to 0.83 | −0.85 to 1.04 | .757 | .846 |
| SDNN | Physical activity | 0.65 | 1.22 | −1.97 to 3.27 | −1.3 to 3.74 | .621 | .339 |
| RMSSD | Intercept | 29.07 | 38.20 | 22.32 to 35.83 | 32.95 to 43.45 | < .001*** | < .001*** |
| RMSSD | Age | −0.44 | −0.26 | −1.64 to 0.76 | −1.2 to 0.67 | .468 | .574 |
| RMSSD | Female (vs. male) | 11.20 | 1.39 | 1.73 to 20.68 | −5.98 to 8.76 | .021* | .707 |
| RMSSD | Other gender | 24.89 | 16.56 | −14.46 to 64.23 | −14.07 to 47.2 | .211 | .285 |
| RMSSD | BMI | 0.24 | 0.12 | −0.9 to 1.38 | −0.76 to 1.01 | .676 | .784 |
| RMSSD | Physical activity | −0.13 | 0.72 | −3.16 to 2.9 | −1.64 to 3.08 | .930 | .544 |

*Comparison of regression coefficients (β) and 95% confidence intervals (CI) for participant characteristics in models predicting HRV metrics from ECG and rPPG measurements. BMI = body mass index. Physical activity refers to self-reported weekly number of days of moderate-to-vigorous exercise. p-values correspond to two-tailed tests. Statistical significance: p < .05*, p < .01**, p < .001***.*

| Metric | Predictor | Scale | Mean Δβ (ms) | 95% CI | % within SESOI | Decision |
| --- | --- | --- | --- | --- | --- | --- |
| RMSSD | Age | per 10 years | 1.7 | [−5.49 to 7.75] | 100.00% | Equivalent |
| RMSSD | Female (vs. male) | per unit | −9.75 | [−15.09 to −4.59] | 97.30% | Equivalent |
| RMSSD | Other gender | per unit | −5.17 | [−13.39 to 0] | 99.00% | Equivalent |
| RMSSD | BMI | per 5 BMI | −0.47 | [−3.21 to 2.65] | 100.00% | Equivalent |
| RMSSD | Physical activity | per active day | 0.81 | [−0.68 to 2.29] | 100.00% | Equivalent |
| SDNN | Age | per 10 years | −1.09 | [−8.12 to 4.51] | 100.00% | Equivalent |
| SDNN | Female (vs. male) | per unit | −6.06 | [−12.64 to −0.14] | 100.00% | Equivalent |
| SDNN | Other gender | per unit | −2.06 | [−10.59 to 3.05] | 100.00% | Equivalent |
| SDNN | BMI | per 5 BMI | 1.39 | [−1.98 to 5.78] | 100.00% | Equivalent |
| SDNN | Physical activity | per active day | 0.52 | [−0.95 −to 2.04] | 100.00% | Equivalent |

*Bootstrapped mean differences in regression coefficients (Δβ, in ms) for participant characteristics in models predicting HRV metrics from ECG and rPPG measurements. Equivalence decisions reflect whether the bootstrapped distribution of differences fell within the smallest effect size of interest (i.e., ±15 ms). CI = confidence interval; BMI = body mass index. All effects are scaled as indicated.*

## Appendix G – Results table: Factors influencing rPPG accuracy

| Fixed Effects | | | | | | |
| --- | --- | --- | --- | --- | --- | --- |
|  | log(*β*) | exp(*β*) | *95% CI* | SE (log) | *t* | *p* |
| Intercept | −0.35 | 0.71 | 0.64 to 0.78 | 0.05 | −6.64 | < .001*** |
| Video length | −0.09 | 0.91 | 0.87 to 0.96 | 0.03 | −3.39 | < .001*** |
| Total movement | 0.28 | 1.33 | 1.2 to 1.46 | 0.05 | 5.52 | < .001*** |
| Proportion of hands visible | 0.29 | 1.33 | 1.07 to 1.65 | 0.11 | 2.62 | .029* |
| Face covered | 0.01 | 1.01 | 0.91 to 1.12 | 0.05 | 0.18 | .86 |
| Skin type (Fitzpatrick) | 0.16 | 1.17 | 1.06 to 1.3 | 0.05 | 3.02 | .003** |
| Luminance | −0.15 | 0.86 | 0.79 to 0.93 | 0.04 | −3.73 | < .001*** |
| Video length × total movement | 0.03 | 1.04 | 0.97 to 1.11 | 0.03 | 1.00 | .316 |
| Video length × hands visible | 0.00 | 1.00 | 0.93 to 1.07 | 0.04 | −0.05 | .957 |
| Video length × face covered | 0.00 | 1.00 | 0.95 to 1.04 | 0.02 | −0.17 | .864 |
| Video length × skin type | 0.00 | 1.00 | 0.95 to 1.05 | 0.02 | 0.02 | .986 |
| Video length × luminance | 0.00 | 1.00 | 0.96 to 1.04 | 0.02 | −0.06 | .953 |
| Movement × hands visible | −0.01 | 0.99 | 0.95 to 1.03 | 0.02 | −0.55 | .58 |
| Movement × face covered | 0.06 | 1.06 | 0.97 to 1.17 | 0.05 | 1.29 | .201 |
| Movement × skin type | −0.02 | 0.98 | 0.89 to 1.09 | 0.05 | −0.31 | .76 |
| Movement × luminance | 0.01 | 1.01 | 0.95 to 1.08 | 0.03 | 0.41 | .681 |
| Hands visible × face covered | −0.17 | 0.84 | 0.66 to 1.07 | 0.13 | −1.39 | .188 |
| Hands visible × skin type | 0.13 | 1.14 | 0.91 to 1.44 | 0.12 | 1.16 | .265 |
| Hands visible × luminance | 0.07 | 1.08 | 0.91 to 1.27 | 0.09 | 0.85 | .404 |
| Face covered × skin Type | −0.04 | 0.96 | 0.87 to 1.05 | 0.05 | −0.87 | .389 |
| Face covered × luminance | −0.01 | 0.99 | 0.93 to 1.06 | 0.03 | −0.24 | .812 |
| Skin type × luminance | −0.10 | 0.91 | 0.84 to 0.98 | 0.04 | −2.57 | .012* |

| Random Effects | | | | |
| --- | --- | --- | --- | --- |
|  |  | Variance | SD |  |
| Participant: | Intercept | 0.12 | 0.35 |  |
|  | Video length | 0.02 | 0.13 |  |
|  | Luminance | 0.05 | 0.22 |  |
|  | Total movement | 0.05 | 0.23 |  |
|  | Proportion of hands visible | 0.20 | 0.44 |  |
| Observations: | 2412 | | | |
| Marginal *R^2^*/ Conditional *R^2^*: | 0.202 / 0.659 | | | |

## Appendix H – Results table: Emotion anticipation task analysis

| Fixed Effects (ECG) | | | | | |
| --- | --- | --- | --- | --- | --- |
|  | *β* | *95% CI* | SE | *z* | *p* |
| Intercept | 0.00− | −0.07 to 0.07−− | 0.04 | 0.09− | .927 |
| Object vs. erotica | −0.01 | −0.06 to 0.05− | 0.03 | −0.29 | .770 |
| Object vs. injury | 0.08 | 0.02 to 0.14− | 0.03 | 2.51 | .012* |
| Component: D1 vs. D2 | 0.26 | 0.19 to 0.33 | 0.04 | 7.48 | < .001*** |
| Component: A1 vs. D1/D2 | 0.62 | 0.56 to 0.68 | 0.03 | 20.49 | < .001*** |
| Component: All vs. PDP | 0.55 | 0.49 to 0.60 | 0.03 | 19.60 | < .001*** |
| Object vs. erotica × D1 vs. D2 | 0.05 | −0.05 to 0.14− | 0.05 | 0.97 | .331 |
| Object vs. injury × D1 vs. D2 | −0.07− | −0.16 to 0.03− | 0.05 | −1.37− | .170 |
| Object vs. erotica × A1 vs. D1/D2 | −0.03− | −0.11 to 0.06− | 0.04 | −0.62− | .535 |
| Object vs. injury × A1 vs. D1/D2 | 0.00− | −0.08 to 0.09− | 0.04 | 0.08− | .934 |
| Object vs. erotica × All vs. PDP | −0.03− | −0.11 to 0.04− | 0.04 | −0.85− | .398 |
| Object vs. injury × All vs. PDP | −0.08− | −0.16 to −0.01−− | 0.04 | −2.14− | .032* |

Note. *β* = standardized coefficient (outcome z-scored using ECG mean and SD). CI = 95% confidence interval. Tests use Wald z-statistics.

| Fixed Effects (rPPG) | | | | | |
| --- | --- | --- | --- | --- | --- |
|  | *β* | *95% CI* | SE | *z* | *p* |
| Predictor | 0.02− | −0.05 to 0.09−− | 0.04 | 0.64− | .523 |
| Intercept | 0.00− | −0.06 to 0.07− | 0.03 | 0.09− | .930 |
| Object vs. erotica | 0.07 | −0.01 to 0.14− | 0.04 | 1.78 | .076 |
| Object vs. injury | 0.18 | 0.10 to 0.26 | 0.04 | 4.53 | < .001*** |
| Component: D1 vs. D2 | 0.74 | 0.67 to 0.80 | 0.04 | 20.90 | < .001*** |
| Component: A1 vs. D1/D2 | 0.45 | 0.38 to 0.51 | 0.03 | 13.50 | < .001*** |
| Component: All vs. PDP | 0.02 | −0.09 to 0.13− | 0.06 | 0.34 | .733 |
| Object vs. erotica × D1/D2 | −0.07− | −0.19 to 0.04− | 0.06 | −1.26− | .209 |
| Object vs. injury × D1 vs. D2 | −0.04− | −0.13 to 0.06− | 0.05 | −0.72− | .470 |
| Object vs. erotica × A1 vs. D1/D2 | 0.01 | −0.09 to 0.11− | 0.05 | 0.18 | .857 |
| Object vs. injury × A1 vs. D1/D2 | −0.04− | −0.13 to 0.06− | 0.05 | −0.78− | .436 |
| Object vs. erotica × All vs. PDP | −0.05− | −0.15 to 0.04− | 0.05 | −1.15− | .251 |

Note. *β* = standardized coefficient (outcome z-scored using ECG mean and SD). CI = 95% confidence interval. Tests use Wald z-statistics.

# References

Bland, J. M., & Altman, D. G. (1999). Measuring agreement in method comparison studies. *Statistical Methods in Medical Research*, *8*(2), 135–160. https://doi.org/10.1177/096228029900800204

Bland, J. M., & Altman, D. G. (2007). Agreement Between Methods of Measurement with Multiple Observations Per Individual. *Journal of Biopharmaceutical Statistics*, *17*(4), 571–582. https://doi.org/10.1080/10543400701329422

Bradski, G. (n.d.). *The OpenCV Library*. Dr. Dobb’s. Retrieved 27 December 2023, from http://www.drdobbs.com/open-source/the-opencv-library/184404319

Fine, J., Branan, K. L., Rodriguez, A. J., Boonya-ananta, T., Ajmal, Ramella-Roman, J. C., McShane, M. J., & Coté, G. L. (2021). Sources of Inaccuracy in Photoplethysmography for Continuous Cardiovascular Monitoring. *Biosensors*, *11*(4), 126. https://doi.org/10.3390/bios11040126

Fitzpatrick, T. B. (1988). The Validity and Practicality of Sun-Reactive Skin Types I Through VI. *Archives of Dermatology*, *124*(6), 869–871. https://doi.org/10.1001/archderm.1988.01670060015008

Giorgino, T. (2009). Computing and Visualizing Dynamic Time Warping Alignments in R: The dtw Package. *Journal of Statistical Software*, *31*, 1–24. https://doi.org/10.18637/jss.v031.i07

Haghayegh, S., Kang, H.-A., Khoshnevis, S., Smolensky, M. H., & Diller, K. R. (2020). A comprehensive guideline for Bland–Altman and intra class correlation calculations to properly compare two methods of measurement and interpret findings. *Physiological Measurement*, *41*(5), 055012. https://doi.org/10.1088/1361-6579/ab86d6

Kartynnik, Y., Ablavatski, A., Grishchenko, I., & Grundmann, M. (2019). *Real-time Facial Surface Geometry from Monocular Video on Mobile GPUs* (arXiv:1907.06724). arXiv. https://doi.org/10.48550/arXiv.1907.06724

Kirk, P. A., Davidson Bryan, A., Garfinkel, S. A., & Robinson, O. J. (2022). RapidHRV: an open-source toolbox for extracting heart rate and heart rate variability [PeerJ]. *PeerJ*. https://doi.org/10.7717/peerj.13147

Lipponen, J. A., & Tarvainen, M. P. (2019). A robust algorithm for heart rate variability time series artefact correction using novel beat classification. *Journal of Medical Engineering & Technology*, *43*(3), 173–181. https://doi.org/10.1080/03091902.2019.1640306

Malik, M., Bigger, J. T., Camm, A. J., Kleiger, R. E., Malliani, A., Moss, A. J., & Schwartz, P. J. (1996). Heart rate variability: Standards of measurement, physiological interpretation, and clinical use. *European Heart Journal*, *17*(3), 354–381. https://doi.org/10.1093/oxfordjournals.eurheartj.a014868

Müller, M. (Ed.). (2007). Dynamic Time Warping. In *Information Retrieval for Music and Motion* (pp. 69–84). Springer. https://doi.org/10.1007/978-3-540-74048-3_4

Munoz, M. L., Roon, A. van, Riese, H., Thio, C., Oostenbroek, E., Westrik, I., Geus, E. J. C. de, Gansevoort, R., Lefrandt, J., Nolte, I. M., & Snieder, H. (2015). Validity of (Ultra-)Short Recordings for Heart Rate Variability Measurements. *PLOS ONE*, *10*(9), e0138921. https://doi.org/10.1371/journal.pone.0138921

O’Neal, W. T., Chen, L. Y., Nazarian, S., & Soliman, E. Z. (2016). Reference Ranges for Short-Term Heart Rate Variability Measures in Individuals Free of Cardiovascular Disease: The Multi-Ethnic Study of Atherosclerosis (MESA). *Journal of Electrocardiology*, *49*(5), 686–690. https://doi.org/10.1016/j.jelectrocard.2016.06.008

Pham, T., Lau, Z. J., Chen, S. H. A., & Makowski, D. (2021). Heart Rate Variability in Psychology: A Review of HRV Indices and an Analysis Tutorial. *Sensors*, *21*(12), Article 12. https://doi.org/10.3390/s21123998

Sakoe, H., & Chiba, S. (1978). Dynamic programming algorithm optimization for spoken word recognition. *IEEE Transactions on Acoustics, Speech, and Signal Processing*, *26*(1), 43–49. https://doi.org/10.1109/TASSP.1978.1163055

Shaffer, F., & Ginsberg, J. P. (2017). An Overview of Heart Rate Variability Metrics and Norms. *Frontiers in Public Health*, *5*. https://doi.org/10.3389/fpubh.2017.00258

Wang, W., Brinker, A. C. den, Stuijk, S., & Haan, G. de. (2017). Robust heart rate from fitness videos. *Physiological Measurement*, *38*(6), 1023. https://doi.org/10.1088/1361-6579/aa6d02

Zhang, F., Bazarevsky, V., Vakunov, A., Tkachenka, A., Sung, G., Chang, C.-L., & Grundmann, M. (2020). *MediaPipe Hands: On-device Real-time Hand Tracking* (arXiv:2006.10214). arXiv. https://doi.org/10.48550/arXiv.2006.10214
